# Mathematical model of the life cycle of taenia-cysticercosis: transmission dynamics and chemotherapy (Part 1)

**DOI:** 10.1186/s12976-018-0090-0

**Published:** 2018-11-19

**Authors:** Marco V. José, Juan R. Bobadilla, Norma Y. Sánchez-Torres, Juan Pedro Laclette

**Affiliations:** 1 Theoretical Biology Group, Instituto de Investigaciones Biomédicas, Universidad Nacional Autónoma de México, Ciudad Universitaria, 04510 CDMX, Mexico; 2 Department of Immunology, Biomedical Research Institute, Universidad Nacional Autónoma de México, Ciudad Universitaria, 04510 CDMX, Mexico

**Keywords:** Taenia-cysticercosis, Mathematical model, Chemotherapeutic interventions, Prevention and control, Susceptible-infected model

## Abstract

**Background:**

*Taenia solium* is the aetiological agent of human taeniasis, pig cysticercosis and human neurocysticercosis, which are serious public health problems, especially in developing countries.

**Methods:**

A mathematical model of the transmission dynamics of taeniasis-cysticercosis is formulated. The model consists of a coupled system of differential equations, which are density-dependent equations for describing the flow of the parasite through the life cycle. The model is hybrid since it comprises deterministic equations with stochastic elements which describe changes in the mean parasite burden and incorporates the overall pattern of the parasites’ distribution.

**Results:**

Sensitivity and bifurcation analyses were carried out to determine the range of values of the model. The model can reproduce the observed epidemiological patterns of human taeniasis, pig and human cysticercosis. For example, for a wide range of parameter values, the mean intensity of adult worms tends to rapidly stabilize in one parasite per individual host. From this model, we also derived a Susceptible-Infected model to describe the prevalence of infection in humans and pigs. Chemotherapeutic interventions against pig cysticercosis or human taeniasis may reduce rapidly and effectively the mean intensity of human taeniasis, pig cysticercosis and human cysticercosis. This effect can be achieved even if the protective efficacy of the drug is of the order of 90% and the coverage rate is 90%. This means that health in humans infected either with adult worms or cysticerci may be achieved by the application of anthelmintic drugs against pig cysticercosis. However, treatment against human cysticercosis alone, does not influence neither human teniasis nor pig cysticercosis. This is because human cysticercosis infection does not influence the value of the basic reproductive number (*Ro*).

**Conclusions:**

Even coverage of 100% in the administration of anthelmintics did not eliminate the infection. Then elimination of the infection in all hosts does not seem a feasible goal to achieve by administering only chemotherapeutic interventions. Throughout the manuscript a discussion of our model in the context of other models of taeniasis-cysticercosis is presented.

**Electronic supplementary material:**

The online version of this article (10.1186/s12976-018-0090-0) contains supplementary material, which is available to authorized users.

## Background

*Taenia solium* cysticercosis constitutes a serious public health problem, especially in developing countries where pigs are raised in rural communities. In developed countries taeniasis-cysticercosis was practically eliminated since the beginning of the twentieth century. It has, however, reappeared in recent years because immigration of a considerable number of people from developing countries who may be carriers of *T. solium* and because of increased world-wide tourism [1–11]. In countries in which this parasitosis remains endemic, cysticercosis may affect 2 to 4% of the general population [7, 10]. It is a source of important economic loss in pig rearing in the Third World [12]. In humans, *T. solium* frequently causes neurocysticercosis (NC) which is one of the main causes of epilepsy in the world [13]. It has been found that 29% of epilepsy in endemic communities are attributable to NC [10], which in some communities affect more than 1% of the population. By conservative estimates, greater than 5 million cases of epilepsy worldwide, which are all preventable, are caused by NC [10]. NC is one of the most frequent parasite infections associated with neurological chronic morbidity in the United States [14]. Human NC evolution is highly variable from complete latency to severe encephalopathy with psychomotor regression. The most frequent invalidating symptoms include seizures, headache, vomiting, endocranial hypertension, confusion, focal pyramidal deficits, cranial nerve dysfunction, cerebellar syndrome, amaurosis (vision loss or weakness) and dementia. It is also a main cause of late onset-epilepsy, accounting for more than 50% of such cases and approximately 30% of all patients develop hydrocephalus secondary to obstruction of cerebroespinal fluid circulation [15].

Pediatric cases of neurocysticercosis have been increasingly diagnosed in the US and other countries [6, 11, 14, 16]. A retrospective series of 47 pediatric cases of NC were reported in a children hospital in Chicago where NC is a relatively common cause of febrile seizures in children who require emergency attention [5]. The high frequency and seriousness of NC justifies the efforts to prevent it. Transmission of the disease was described over a century ago and is clearly related with rustic rearing of pigs in impoverished sectors of the rural population. There is evidence that rustically reared pigs become infected within the first days after birth [17]. The requirement of the pig as an obligatory intermediate host for the maintenance of the *T. solium* life cycle may allow the interference with transmission by modifying pig exposure to cysticercosis. This possibility is of importance considering that in most cases, pigs live no more than a year.

Since pig is an indispensable intermediate host, transmission could be reduced by lowering the prevalence of pig cysticercosis through effective mass chemotherapy and/or vaccination. However, application of control measures demands the identification of high prevalence areas and, the availability of reliable, sensitive and, specific diagnostic procedures [18]. More recently, TSOL18 vaccine has proven to be the most effective vaccine against *T. solium*, with independent experimental vaccine trials carried out in Mexico, Peru, Cameroon and Honduras inducing 99.3–100% protection against an experimental challenge infection with *T. solium* eggs in pigs [19–23].

Numerous mathematical models have been elaborated about the transmission dynamics of several infectious diseases produced by helminths [24–31]. Several mathematical models of cestode infections such as *E. granulosus*, *Taenia hydatigena* and *Taenia ovis* in sheep and dogs have been developed [32–34]. Considerable advances have been made in breaking the “epidemiological code” of the family taeniidae with the aid of mathematical modelling. This family contains such zoonotic parasites as *Echinococcus granulosus, Echinococcus multilocularis, Taenia solium* and *Taenia saginata.* A preliminary model for the dynamics of cestode infections was proposed by Keymer [31]. This model described the dynamics of the larval, adult and egg populations for several tapeworm species. The model was analysed and shown to obey a threshold criterion: when a combination of parameters called the basic reproductive number, usually denoted by *Ro*, exceeded 1, the model suggested that the parasite population would, over time, reach a stable equilibrium level. When the basic reproductive number was lower than the needed value for the maintenance of the parasite population, it became extinct over time. Keymer and Anderson [35] used a model to describe the dynamics of the rat tapeworm *Hymenolepis diminuta*, which may infect man through accidental ingestion of the intermediate host, beetles of the *Tribolium* species. They suggested that similar models could be used to describe the dynamics of parasites such as *Echinococcus granulosus* and *Diphyllobotrium latum*.

Much of the current theory of helminth infections is based on the pioneering work of Kostitsyne [36], who formulated a deterministic model consisting of an infinite series of differential equations describing changes in the densities of hosts harbouring any specified number of parasites. The major contribution of this early study was the recognition that the classical epidemic models [37–40] were inappropriate descriptions of the dynamics of helminth parasites. Models of helminth transmission must take account of the fact that the pathology induced by parasitic infection, the fecundity, mortality and establishment of the parasites, and the host responses generated by infection all typically depend on the number or burden of parasites harboured by the host.

However, Susceptible-Infected-Recovered/Immune (SIR/I) models, suitable for microparasitic infections (i.e. bacteria, viruses and protozoa), have been formulated to capture the transmission dynamics of the macroparasite *T. solium* [41–43]. The SIR/I model ignores the life cycle of the helminth parasite. It is assumed that all individuals are in contact with one another randomly. Explicitly, these models assume that the number of infected individuals is equal to the number of susceptibles hosts times the probability of escaping infection due to a contact with one infected individual [41–43]. Kyvsgaard et al. [41], Winskill et al. [42], and Braae et al. [43] assumed in their equations that the rate of transmission (*β*) obeys the mass action principle, i.e. the rate of transmission is proportional to the product of the density of susceptible *X* times the density of infected individuals *Y* (*βXY*). The symmetry of the mass action incidence theoretically allows that susceptible individuals can transmit susceptibility [44]. When this happens, the infections have a SIS-type spread in human populations.

For microparasitic infections, the basic reproductive number, *Ro*, is defined as the average number of secondary infections produced when one infected individual is introduced into a host population where everyone is susceptible. The question of how to measure the number of new infected individuals in the case of persistent helminth infections in which the hosts (definitive and intermediate) are continually re-infected cannot be addressed by SIR models. SIR models do not consider reinfections and the fact that the distribution of most helminth infections is overdispersed (the variance greatly exceeds the mean) [45, 46]. The negative binomial distribution has been found to provide a good empirical description of the distribution of most helminth parasites [47–49]. For many deterministic epidemiology models, an infection can get started in a fully susceptible population if and only if *Ro* > 1, thus the basic reproduction number *Ro* is considered as the threshold quantity that determines when an infection can invade and persist in a new host population.

Helminth infections, in contrast, are typically endemic and *Ro* is defined as the average number of female offspring (in the case of a dioecious species) produced throughout the expected lifespan of a reproductively mature female parasite (which attains reproductive maturity in the absence of density-dependent constraints on population growth). *T. solium* is hermaphrodite and its mating probability is therefore equal to 1 [26–29].

In this work, we develop, for the first time, a density-dependent model for the transmission dynamics of taeniasis-cysticercosis. The model captures the flow of the parasite throughout its life cycle. It explicitly considers the overdispersed distribution of the parasite in both pigs and humans. It is expressed in terms of the mean worm intensity and prevalence of infection. This model will permit to realistically interrogate the transmission dynamics of the taeniasis-cysticercosis and to understand the effects of chemotherapeutic and vaccination interventions. The article is being published in two parts. The first part is organized as follows. We present and analyse the empirical evidence of the epidemiology of taeniasis-cysticercosis together with a detailed description of the life cycle. Second, we present a derivation of the mathematical model of the transmission dynamics of the life cycle of taeniasis-cysticercosis. The dynamical properties of this model are examined and bifurcation analyses of some of its parameters are presented. Third, computer simulation experiments (CSE) of this model are carried out to answer several questions such as the likely impact of different chemotherapeutic strategies aimed to interrupt transmission. Modifications of the density-dependent model lead to a new compartmental model which describes the prevalence of infection in pigs and humans. This Susceptible-Infected (SI) model, permits to interrogate the actual transmission dynamics of the taeniasis-cysticercosis and to understand the effects of chemotherapeutic or vaccination interventions. With the density-dependent model, we present different drug interventions against pig cysticercosis, whilst with the compartmental model we simulate chemotherapeutic interventions against human taeniasis. We explore different rates of coverage and different rates of efficacies for the corresponding anthelmintic. The second part of this article deals with the extension of the SI model to evaluate both chemotherapeutic and vaccination interventions. Finally, the results are discussed in terms of the mathematical theory of infectious diseases and in terms of different chemotherapeutic interventions that can be routinely implemented against either swine cysticercosis and/or human taeniasis.

## Methods

### Life cycle

**Fig. 1:**
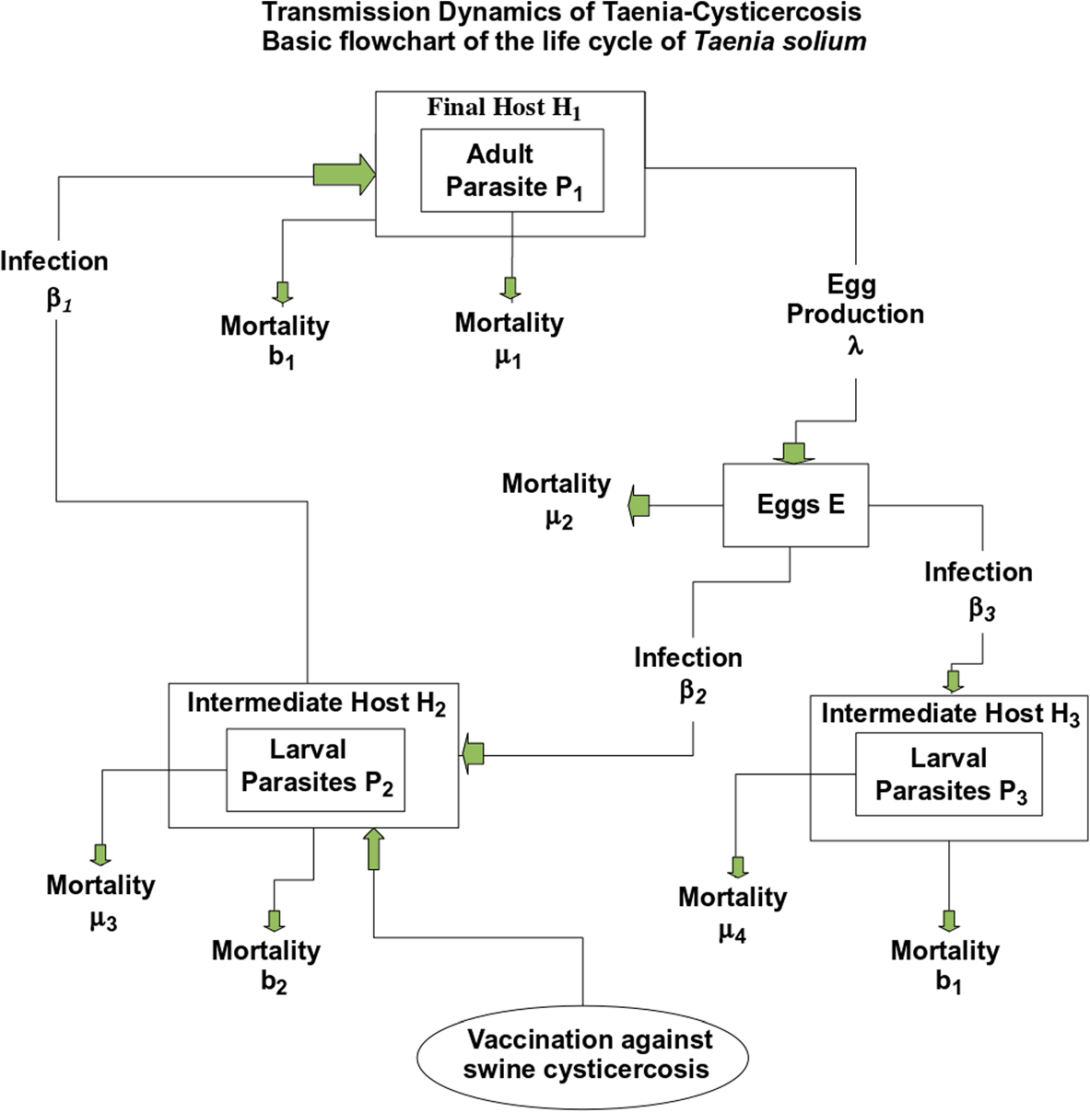
Transmission dynamics of taenia-cysticercosis. Basic flowchart of the life cycle of the tapeworm *Taenia solium*

*T. solium*, the pork tapeworm, is a cestode that inhabits the intestinal lumen of humans. Humans are the only known definitive host of *T. solium*. The life cycle of *T. solium* includes a larval phase (cysticercus) which affects both the human being and the pig (intermediate host). NC develops when the cysticerci infect the nervous system. Taeniasis, i.e., general infection with *T. solium*, is acquired when humans ingest undercooked pork that is infected viable cysticerci. After the cysticercus is ingested, the larva evaginates from the cyst and anchor in the intestinal wall and develops into a sexually mature adult worm. Each day a few mature proglottides are detached from the distal end of the mature worm, reaching the environment mixed into the faecal material. Each gravid proglottide contains around 60,000 eggs [50]. In areas with deficient sanitation, eggs may contaminate vegetables and food. When humans and pigs ingest these eggs, cysticercosis will develop, thus completing the life cycle of the parasite (see Fig. 1).

### Prevalence of taeniasis and distribution of adult worms

In helminth infections, it is common to measure the mean parasite load. This statistic implies that one individual may harbour one or several adult worms. The dynamical properties of different mating behaviours of helminth parasites and their “degree of togetherness” have been extensively studied [51]. However, a typical signature of the infection of *T. solium* is that a single adult worm occurs in each infected individual. However, multiple worm infections have been detected in an individual with at least 7 *T. solium* tapeworms [52]. *T. solium* is hermaphroditic and prefers the method of self-fertilization and the parasite may grow in size by increasing its number of proglottides. Then in the case of *T. solium* we can talk of a “degree of loneliness”. *Taenia solium* taeniasis tends to have a low prevalence, typically of ≤ 1% even in endemic communities [52], and a community with a prevalence of ≥ 1% is considered hyperendemic [53]. The prevalence of taeniasis in Honduras has been reported to be 2.5% [54] and in Guatemala 2.7% [52]. The overall seroprevalence of antibodies to *T. solium* in Honduras was 17% suggesting high transmission rates [54]. It has been reported that individuals with taeniasis are geographically clumped [55]. The presence of individuals with intestinal infections of *T. solium* in the immediate environment increases an individual risk of developing *T. solium* cysticercosis [56]. This has been demonstrated in endemic rural communities [56–60]. The relation between the prevalence of infection, *P* the degree of aggregation of a helminth parasite, *k* (where *k* represents inversely the degree of aggregation of the parasite) and, the mean intensity of the infection, *M* can be described by [51],

1 $$ P=1-{\left(1+\frac{M}{k}\right)}^{-k}. $$

**Fig. 2:**
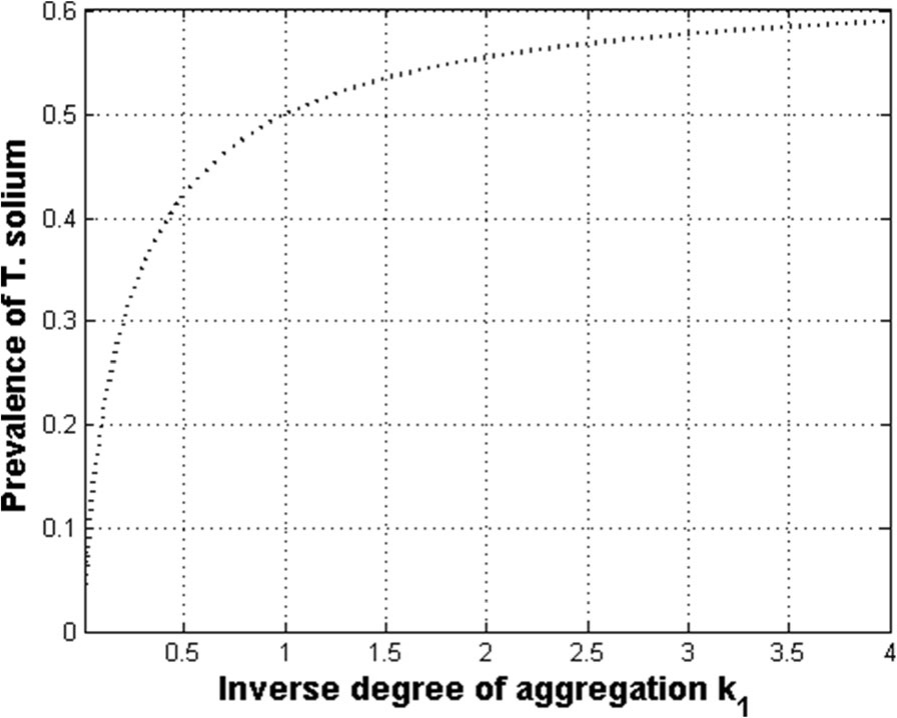
Relationship between the prevalence of infection (*P*), and the degree of worm aggregation (*k*) when the mean intensity (*M*) is equal to one in all individuals

If the parameter *k* tends to infinity the frequency distribution of parasites collapses to a Poisson distribution and helminth parasites are independently randomly distributed. As *k* tends to zero the worms are severely aggregated. Equation (1) is illustrated in Fig. 2 when *M* is equal to 1. Given the constraint of one adult worm per individual there should be only one hyperbolic curve along which the relationship among prevalence of infection, mean intensity and the degree of aggregation can be uniquely described. The hyperbolic pattern means that when human hosts eat infected pork meat, they are sampling larvae from an overdispersed distribution. Since the mean worm intensity starts from values close to zero and increase rapidly to 1, then there is a period in which individuals with several larvae reflect the overdispersed distribution until, in average, each infected individual harbour one adult parasite. It has been observed that mean prevalence increases with age until the 30–39-year age group and declined thereafter [48]. Note that when the value of *k* is very large the theoretical prevalence of the infection is at most 60% when *M* = 1 in the 100% of the cases. When *M* is greater than 1, in at least a small percentage (say 7–14%) of the cases, the prevalence of the infection may approach rapidly to 100% (not shown). This is consistent with the observation that very few individuals with taeniasis are found even in endemic regions with cysticercosis. Since typical values of the prevalence of infection of taeniasis are at most of the order of 1 to 3% then, the degree of aggregation should be of the order of 0.02 or less and at most of the order of 0.2 (see the value of *k*_1_ in Table 1). The great difficulty in finding adult worms, even in places of very high prevalence of pig cysticercosis (5 to 20%), is undoubtedly a very common and one of the most intriguing experiences in the field.

### Distribution of cysticerci in experimentally and naturally infected pigs

In the case of cestodes the negative binomial has been useful to describe the distribution of *H. diminuta* in the intermediate host in both laboratory and field populations [35, 60]. In addition, the negative binomial can also be used to describe the distribution of adult tapeworms in the definitive host (e.g. *Caryophyllaeus laticeps* in the bream, *Abrams brama*) and also larval tapeworms in the second intermediate host (e.g. *Schistocephalus solidus* in the stickleback *Gasterosteus acuelatus* [61]. Frequency distributions of cysticerci in loin, intercostal, leg, shoulder, tongue, diaphragm, and masseter muscle of 41 experimentally infected pigs have been reported [18]. All the estimated values of the parameter *k*_2_ (see Table 1) fell in the range of 0.23–0.37 indicating a high and a similar degree of cysticercus aggregation, i.e., most of the pigs harbour few parasites as opposed to few pigs with most of the parasites, regardless of the location of the parasite. The degree of aggregation of cysticerci in the population of pigs therefore can be estimated either by sampling a specific part of the pig or by sampling the whole pig [18].

## Density-dependent model

To elaborate the mathematical model, three distinct developmental stages should be considered: the adult worm in the definitive host, the free-living parasite egg and, the larval worm in the intermediate host.

### Infection of the intermediate host (pigs)

Infection of the intermediate host occurs because of ingestion of free-living infective stages. A functional response of the host to parasite density may be of significance, since tapeworms are unusual among economically important parasites in that all transmission links are effectively governed by predator-prey associations. Here we assume that the intermediate host acquires parasites at a rate proportional to the density of the hosts, *H*_2_, and the density of infective parasite eggs, *E*. If *β*_2_ represents the rate of transmission, and if the average prepatent period (the time period from entry into the intermediate host to the development of the stage infective to the definitive host) is *T*_2_ time units, then the net rate of parasite acquisition is *β*_2_*H*_2_*E*(*t* − *T*_2_). Of those parasites acquired by an individual host, only a proportion, *D*_2_ survive to reach infectivity, then

$$ {D}_2=\exp \left[-{T}_2\left({\mu}_3+{b}_2\right)\right], $$

where *μ*_3_ is the rate of mortality of larval parasites during the prepatent period and, *b*_2_ is the rate of loss of larval parasites during the prepatent period due to natural host mortalities.

### Natural larval parasite mortality

Little is known about the survival of larval tapeworms in the intermediate host. Here we can assume that the per capita rate of natural larval mortality ${\mu}_3^{\ast }(i),$ is linearly related to larval burden *i*, such that

$$ {\mu}_3^{\ast }(i)={\mu}_3+{\alpha}_2i, $$

where 1/*μ*_3_ represents the expected life span of larval parasites in the absence of density-dependent constraints and *α*_2_ is a coefficient measuring the severity of density dependent constraints on larval survival. Thus, the net rate of larval losses in the population is

$$ {H}_2\sum \limits_{i=0}^{\infty}\left({\mu}_3+{\alpha}_2i\right){ip}_2(i)={H}_2\sum \limits_{i=o}^{\infty }{\mu}_3^{\ast }(i){ip}_2(i), $$

where *p*_2_(*i*) represents the probability that an individual host contains *i* parasites.

### Loss of larval parasites due to intermediate host mortality

Intermediate hosts (pigs) are assumed to die at a per capita rate *b*_2_ such that the net loss of parasites due to intermediate hosts mortality is

$$ {b}_2{H}_2\sum \limits_{i=o}^{\infty }{ip}_2(i). $$

In the pigs, we can assume that there are detrimental parasite-induced effects on host survival or on host fecundity. In the case of pigs, however, it is unlikely that the pigs would survive long enough in the natural environment for parasite-induced effects to be of significance.

### Loss of larval parasites due to final host infection

Final host infection is achieved by means of a predator-prey association, between definitive and intermediate hosts (to eat raw meat). It is unlikely that the intermediate host density (of pigs) reaches high enough levels to cause a reduction in the infection rates due to satiation or handling time effects, especially since the intermediate host species is not the primary or the only food source of the definitive host. Ingestion is thus assumed to be directly proportional to the density of final and intermediate hosts, denoted by *H*_1_ and *H*_2,_ respectively, such that the net rate of loss to the larval parasite population is

$$ {\beta}_1{H}_1{H}_2\sum \limits_{i=0}^{\infty }{ip}_2(i), $$

where *β*_1_ is a coefficient representing the rate of transmission after consumption of infected meat, and *p*_2_(*i*) is the probability that an individual host harbours *i* parasites.

### Final host infection: the acquisition of mature parasites

Ingestion of an infected intermediate host by a potential definitive host is assumed to be directly proportional to their densities, *H*_1_ and *H*_2,_ such that the net rate of acquisition of mature parasites is

$$ {\beta}_1{H}_1{H}_2\sum \limits_{i=0}^{\infty }{ip}_2(i). $$

Of those parasites acquired by an individual host, only a proportion *D*_1_ survive to reach sexual maturity, since some are lost due to parasite and host mortalities during the prepatent period *T*_1_ (the time period from entry into the definitive host to reproductive maturity when worms begin egg production). If the average length of the prepatent period in the definitive host is *T*_1_ time units, then,

$$ {D}_1=\exp \left[-{T}_1\left({\mu}_1+{b}_1\right)\right], $$

where *μ*_1_ and *b*_1_ are the instantaneous per capita rates of adult parasite and final host mortalities, respectively. Then the net rate of gain of sexually mature adult parasites is,

$$ {D}_1{\beta}_1{H}_1{H}_2\sum \limits_{i=0}^{\infty }{ip}_2(i)\left(t-{T}_1\right), $$

where here and further the term of the sum means evaluating ∑*ip*(*i*) at (*t* − *T*).

### Natural adult parasite mortality

The rate of natural adult parasite mortality is assumed to be dependent on the density of mature parasites within any given individual host. Now and for simplicity density-dependent constraints that affect the fecundity and establishment of the parasites will not be considered. The per capita rate of natural parasite mortality ${\mu}_1^{\ast },$ is assumed to be linearly related to parasite burden *i*, such that,

$$ {\mu}_1^{\ast }={\mu}_1+{\alpha}_1i, $$

where 1/*μ*_1_ represents the expected life span of mature parasites in the absence of density-dependent constraints and *α*_1_ is a coefficient measuring the severity of density-dependent constraints on worm survival. Thus, the net rate of parasite losses in the populations is,

$$ {H}_1\sum \limits_{i=0}^{\infty}\left({\mu}_1+{\alpha}_1\right){ip}_1(i)={H}_1\sum \limits_{i=0}^{\infty }{\mu}_i^{\ast }{ip}_1(i). $$

### Adult parasite losses due to definitive host mortality

Human hosts are assumed to have a constant, instantaneous per capita mortality rate, *b*_1_ such that the net loss of parasites due to host mortality is,

$$ {b}_1{H}_1\sum \limits_{i=0}^{\infty }{ip}_1(i). $$

Adult tapeworms are often considered to be without serious pathogenic effects on the definitive host [62, 63], and here it is assumed that there is no parasite-induced host mortality.

### The production of infective eggs

Most cestodes are hermaphrodite and are thought in general to be able to carry out both self- and cross-fertilization. Now, it seems safe to assume that all worms in the definitive host are equally capable of producing eggs, whether present singly or in multiple worm burdens. If the instantaneous per capita rate of egg production, *λ*_1_, is constant and independent of worm age or density, then the net rate of egg production is equal to *λP*_1_, where *P*_1_ is the number of adult parasites. This is in contrast with dioceious helminths such as hookworms and schistosomes, where the production of viable eggs does not occur unless the worms are mated within the host. The mating function in those parasites (i.e. the probability that any given female worm has been mated) has important consequences with respect to the overall dynamics of the host-parasite interaction [51]. In this model of taeniasis-cysticercosis it will be considered that the mating function is equal to unity since the eggs of some cestode species are immediately infective to the intermediate host, i.e., there are no significant time delays in egg maturation [45].

### Natural mortality of infective eggs

The population of infective eggs is subject to considerable mortality while in the external environment [64, 65]. Assuming that the instantaneous rate of mortality is *μ*_2_ per egg per unit time, the net rate of loss is equal to *μ*_2_*E*, where *E* is the density of infective parasite eggs. Each egg thus has an expected life span of 1/*μ*_2_ .

### Loss of infective stages due to host infection

Infective eggs are removed from the population because of ingestion of potential intermediate hosts (pigs and humans). The rate of loss is equal to *β*_2_*H*_2_*E* + *β*_3_*H*_3_*E* (pigs plus humans), where *H*_3_ is the density of humans infected with eggs that may develop into cysticerci. Loss of eggs as a result of ingestion by animals unsuitable as intermediate hosts is assumed to be incorporated into the term denoting egg mortality (*μ*_2_). The 3 loss terms represent deaths due to natural mortalities (*μ*_2_*E*), losses due to infection of pigs (*β*_2_*H*_2_*E*), and losses due to infection of humans (*β*_3_*H*_3_*E*).

### The distribution of parasite numbers per host

Let *P*_1_= number of adult parasites (taenias), *P*_2_= number of larval parasites (cysticerci) and, *E*= number of tapeworm eggs. Thus, the change of *P*_1_ as a function of time is,

$$ \frac{d{P}_1(t)}{dt}=\mathrm{acquisition}\kern0.34em \mathrm{of}\kern0.34em \mathrm{mature}\kern0.34em \mathrm{parasite}\mathrm{s}\left(\mathrm{definitive}\kern0.34em \mathrm{host}\kern0.34em \mathrm{infection}\right)-\mathrm{natural}\kern0.34em \mathrm{adult}\kern0.34em \mathrm{parasite}\mathrm{mortality}-\mathrm{adult}\kern0.34em \mathrm{parasite}\kern0.34em \mathrm{losses}\kern0.28em \mathrm{due}\kern0.28em \mathrm{to}\kern0.34em \mathrm{definitive}\kern0.34em \mathrm{host}\kern0.34em \mathrm{mortality}, $$

this is,

2a $$ \frac{d{P}_1(t)}{dt}={\beta}_1{H}_1{H}_2{D}_1\sum \limits_{i=0}^{\infty }{ip}_2(i)\left(t-{T}_1\right)-{H}_1\sum \limits_{i=o}^{\infty }{\mu}_1^{\ast }(i){ip}_1(i)-{b}_1{H}_1\sum \limits_{i=0}^{\infty }{ip}_1(i). $$

The change of *P*_2_ as a function of time is,

$$ \frac{d{P}_2(t)}{dt}=\mathrm{infection}\kern0.34em \mathrm{of}\kern0.34em \mathrm{the}\kern0.34em \mathrm{intermediate}\kern0.34em \mathrm{host}-\mathrm{natural}\kern0.34em \mathrm{larval}\kern0.34em \mathrm{parasite}\kern0.34em \mathrm{mortality}-\mathrm{loss}\kern0.34em \mathrm{of}\kern0.34em \mathrm{larval}\kern0.34em \mathrm{parasite}\mathrm{s}\kern0.28em \mathrm{due}\kern0.28em \mathrm{to}\kern0.34em \mathrm{intermediate}\kern0.34em \mathrm{host}\kern0.34em \mathrm{mortality}-\mathrm{loss}\kern0.34em \mathrm{of}\kern0.34em \mathrm{larval}\kern0.34em \mathrm{parasite}\mathrm{s}\kern0.28em \mathrm{due}\kern0.28em \mathrm{to}\kern0.34em \mathrm{definitive}\kern0.34em \mathrm{host}\kern0.34em \mathrm{infection}, $$

this is,

2b $$ \frac{d{P}_2(t)}{dt}={\beta}_2{D}_2{H}_2E\left(t-{T}_2\right)-{H}_2\sum \limits_{i=0}^{\infty}\left({\mu}_3+{\alpha}_2i\right){ip}_2(i)-{b}_2{H}_2\sum \limits_{i=0}^{\infty }{ip}_2(i)-{\beta}_1{H}_1{H}_2\sum \limits_{i=0}^{\infty }{ip}_2(i). $$

The change of *E* as a function of time is,

$$ \frac{dE(t)}{dt}=\mathrm{egg}\kern0.28em \mathrm{production}-\mathrm{natural}\kern0.34em \mathrm{mortality}\kern0.34em \mathrm{of}\kern0.34em \mathrm{infective}\kern0.34em \mathrm{eggs}-\mathrm{loss}\kern0.34em \mathrm{of}\kern0.34em \mathrm{infective}\kern0.34em \mathrm{stages}\kern0.28em \mathrm{a}\mathrm{s}\kern0.28em \mathrm{a}\kern0.28em \mathrm{consequence}\kern0.34em \mathrm{of}\kern0.34em \mathrm{ingestion}\kern0.28em \mathrm{by}\kern0.28em \mathrm{potential}\kern0.34em \mathrm{intermediate}\kern0.34em \mathrm{hosts},\mathrm{this}\kern0.34em \mathrm{is}, $$

2c $$ \frac{dE(t)}{dt}=\lambda {P}_1-{\mu}_2E(t)-{\beta}_2{H}_2E\left(t-{T}_2\right)-{\beta}_3{H}_3E\left(t-{T}_3\right), $$

where *T*_3_ is the time period from entry into the human hosts to the development of cysticerci infective to the intermediate host. Considering that ${\mu}_i^{\ast }(i)={\mu}_1+{\alpha}_1i,$ ${\mu}_3^{\ast }(i)={\mu}_3+{\alpha}_2i,$ and 〈*i*〉_1_ = *P*_1_/*H*_1_, 〈*i*〉_2_ = *P*_2_/*H*_2_, where 〈*i*〉_1_ is first moment of the negative binomial distribution *p*(*i*), and that in general,

$$ \left\langle i\right\rangle =P/H=\sum \limits_{i=0}^{\infty } ip(i) $$

and,

$$ \left\langle {i}^2\right\rangle =P/H+{P}^2\left(k+1\right){H}^2k=\sum \limits_{i=0}^{\infty }{i}^2p(i), $$

where 〈*i*^2^〉 is the second moment of the negative binomial distribution *p*(*i*), and the parameter *k* is the clumping parameter.

Eqs. (2a), (2b) become,

3a $$ \frac{d{P}_1(t)}{dt}={\beta}_1{H}_1{D}_1{P}_2\left(t-{T}_1\right)-{P}_1(t)\left({b}_1+{\mu}_1+{\alpha}_1\right)-\frac{\alpha_1{P}_1^2(t)\left({k}_1+1\right)}{H_1{k}_1} $$

3b $$ \frac{d{P}_2(t)}{dt}={\beta}_2{D}_2{H}_2E\left(t-{T}_2\right)-{P}_2(t)\left({\mu}_3+{b}_2+{\alpha}_2+{\beta}_1{H}_1\right)-\frac{\alpha_2{P}_2^2(t)\left({k}_2+1\right)}{H_2{k}_2} $$

### Time scales

In common with many other parasitic organisms, the expected life spans of the majority of mature and larval tapeworms are many orders of magnitude greater than that of the infective egg. The dynamics of the infective egg population thus operate on a much faster time scale than the dynamics of the other two parasite populations. In addition, the developmental time delays *T*_1_, *T*_2_, and *T*_3_ are generally short in relation to the life span of the hosts and parasites. This suggests that the prepatent time delays are unlikely to be of significance to the dynamics of the parasite population viewed over many generations. An exception to both these generalities is found in the life cycle of *E. granulosus* [33].

### Dynamical properties of the model

Let’s define the mean worm burden as *M* = *P*/*H*. Since the life span of the parasite egg is much shorter than the several months or years of the survival period of the adult worm, it may be assumed that the dynamics of egg population is effectively at equilibrium when compared with that of the host and larval and adult parasite population. This means that Eq. (2c) is equalled to zero. Since age-chemotherapeutic schedules will be examined later then the dynamics will be finally expressed in terms of age. For the sake of simplicity, we are not yet considering the time-dependent behavior of the dynamics. There are other models of helminth infections that include both age and time [66, 67]. The age-time partial differential version of our model is an ongoing work in our group. Additionally, the available population data of helminth infections is typically given in terms of age-intensity and age-prevalence curves [25, 26, 29].

In summary, a system of two non-linear ordinary differential equations that describe the mean intensity of taeniasis in humans (*M*_1_) and the mean intensity of cysticerci in the pigs (*M*_2_) as a function of age, denoted by *a*, are:

4a $$ \frac{d{M}_1(a)}{da}={\beta}_1{D}_1{H}_2{M}_2(a)-\left({b}_1+{\mu}_1+{\alpha}_1\right){M}_1(a)-\frac{\alpha_1\left({k}_1+1\right){M}_1^2(a)}{k_1} $$

4b $$ \frac{d{M}_2(a)}{da}=\frac{\beta_2{D}_2\lambda {H}_1{M}_1(a)}{\mu_2+{\beta}_2{H}_2+{\beta}_3{H}_3}-\left({\mu}_3+{b}_2+{\alpha}_2+{\beta}_1{H}_1\right){M}_2(a)-\frac{\alpha_2\left({k}_2+1\right){M}_2^2(a)}{k_2} $$

### Dynamics of humans with cysticercosis

It is assumed that humans acquire larval parasites (metacestodes) at a rate proportional to the density of hosts, *H*_3_, and the density of infective parasite eggs, *E*. The net rate of parasite acquisition is then *β*_3_*H*_3_*E*. Of those parasites acquired by an individual host, only a proportion, *D*_3_, survive to reach infectivity. If the average prepatent period in the intermediate host is *T*_3_ time units then

$$ {D}_3=\exp \left[-{T}_3\left({\mu}_4+{b}_1\right)\right], $$

where *μ*_4_ and *b*_1_ represent losses of larval parasites during the prepatent period due to natural parasite mortality and natural host mortality, respectively. The net rate of gain to the population of infective larval parasites is *β*_3_*D*_3_*H*_3_*E*(*t* − *T*_3_).

### Natural larval parasite mortality

It is assumed that larval parasites have a constant per capita instantaneous mortality rate, *μ*_4_, independent of either their density or age. In a population of *P*_3_, larval parasites, the net loss of parasites is *μ*_4_*P*_3_. If we consider a natural larval parasite mortality we can assume that this mortality will depend upon the density of larval parasites within individual hosts. Survival and fecundity may be density dependent.

### Loss of larval parasites due to intermediate host mortality

Intermediate hosts are assumed to die at a per capita rate *b*_1_,such that the net rate of loss of parasites is

$$ {b}_1{H}_3\sum \limits_{i=0}^{\infty }{ip}_3(i), $$

where *P*_3_(*i*) represents the probability that an individual host contains *i* parasites. The intermediate human host survives long enough for parasite-induced effects to be of significance mainly in terms of morbidity and mortality.

Ingestion of humans by humans does not exist (or at least cannibalism is not presently a standard route of transmission). Transmission of cysticercosis or taeniasis by host-to-host contact, do not occur, as it has been assumed in other models [41–43].

The severities of density-dependent constraints are presumably due to the action of the immune system. Assuming that *α*_3_ is a coefficient measuring the severity of density- dependent constraints on worm survival, the transmission dynamics of cysticerci in humans can be expressed as

$$ \frac{d{P}_3(t)}{dt}={\beta}_3{D}_3{H}_3E\left(t-{T}_3\right)-\left({\mu}_4+{\alpha}_3\right){P}_3(t)-{b}_1{H}_3\sum \limits_{i=0}^{\infty }{ip}_3(i). $$

Considering the egg population in equilibrium and 〈*i*〉_3_ = *P*_3_/*H*_3_ = *M*_3_, this equation in terms of age becomes,

4c $$ \frac{d{M}_3(a)}{da}=\frac{\beta_3{D}_3\lambda {H}_3{M}_1(a)}{\mu_2+{\beta}_2{H}_2+{\beta}_3{H}_3}-\left({\mu}_4+{b}_1+{\alpha}_3\right){M}_3(a). $$

In summary, Eqs. (4a), (4b), and (4c) represent a mathematical model of the transmission dynamics of cysticercosis which is a coupled system of three non-linear ordinary differential equations. The stability analysis of the model is presented in Additional file 1.

### The basic reproduction number (*Ro*)

*Ro* is defined as the number of female offspring which are produced in average by one female parasite throughout her reproductive life span and which themselves survive to achieve sexual maturity in a population of *N* uninfected hosts, in other words, in the absence of density dependent constraints on worm survival. Thus *Ro* determines, in part, the level of parasitism in the population. Conceptually, *Ro* can be viewed as,

$$ Ro=\frac{Factors\kern0.5em of\kern0.5em the\kern0.5em transmission\kern0.5em of\kern0.5em the\kern0.5em parasite}{Mortality\kern0.5em of\kern0.5em the\kern0.5em adult,\kern0.5em larvae\kern0.5em and\kern0.5em egg}. $$

In our model, it can easily be shown that,

5 $$ Ro=\frac{\lambda {H}_1{H}_2{\beta}_1{\beta}_2{D}_1{D}_2}{\left({\mu}_3+{b}_2+{\alpha}_2+{\beta}_1{H}_1\right)\left({\mu}_2+{\beta}_2{H}_2+{\beta}_3{H}_3\right)\left({b}_1+{\mu}_1+{\alpha}_1\right)}. $$

### Data sources and units of the parameters

In Table 1 the ranges of values of 24 parameters of the model are shown. The meanings and units of variables and parameters are enlisted in Table 2. The ranges of values of 11 parameters are taken from the literature [18, 52, 58–65, 68] and the ranges of 5 are derived in this paper (*k*_1_, *D*_1_, *D*_2_, *D*_3_, *T*_3_). The values of the demographic parameters *b*_1_ and *b*_2_ are typical of rural communities. Then we have 3 transmission coefficients (*β*_1_, *β*_2_, *β*_3_) and 3 density-dependent constraints (*α*_1_, *α*_2_, *α*_3_) for which bifurcation analyses are presented in the next section. For simplicity, we assume that *H*_3_ = *H*_1_. Gemmel [62] pointed out that quantitative information regarding the values of several biological parameters were needed to quantify the transmission dynamics of *T. solium*. With the present sensitivity analysis and the bifurcation diagrams, we have uncovered the ranges of all parameters of the life cycle. This is particularly relevant for the values assigned to the transmission coefficients, *β*_1_, *β*_2_, and *β*_3_ which are expressed in terms of year^− 1^, and the severities of the density-dependent constraints *α*_1_, *α*_2_, and *α*_3_ whose units are given as number of parasite losses per year ^− 1^ per parasite burden. The time units of the prepatent periods (*T*_1_, *T*_2_, *T*_3_) are in terms of year and all the per capita rates of mortality (*μ*_1_, *μ*_2_, *μ*_3_, *μ*_4_, *b*_1_, *b*_2_), are per year ^− 1^; the density units of the hosts (*H*_1_, *H*_2_, *H*_3_) are in terms of the number of individuals per square kilometer; the rate of fecundity (*λ*) is in terms of the number of eggs per worm per year.

**Table 1:** Parameters of the Mathematical Model Of Taenia/Cysticercosis

Parameter	Symbol	Estimate	Source
Inverse measure of aggregation of *T. solium*	*k*_1_	0.02–0.2	This work
Inverse measure of aggregation of cysticerci	*k*_2_	0.23–0.37	[18]
Rate of transmission of adult parasites	*β*_1_	0.1–0.36	This work
Rate of transmission of larvae to pigs	*β*_2_	0.35–1	This work
Rate of transmission of larvae to humans	*β*_3_	0.036–0.1	This work
See text for definition	*D*_1_	0.9–1	
See text for definition	*D*_2_	0.6–0.9	
See text for definition	*D*_3_	0.9–1	
See text for definition	*T*_1_	0.16–0.33	[14]
See text for definition	*T*_2_	0.208	[60]
See text for definition	*T*_3_	0.3–0.67	
Density of humans with T. solium (*H*_1_) or with cysticerci (*H*_3_)	*H*_1_ or *H*_3_	0.001–0.01	[48, 54, 67]
Density of hosts with cysticerci (pigs)	*H*_2_	0.1–0.6	[62]
Rate of mortality of the definitive host	*b*_1_	0.016	
Rate of mortality of the intermediate host	*b*_2_	0.8–1.5	
Severity of density-dependent constraints (taenia)	*α*_1_	0.001–0.01	This work
Severity of density-dependent constraints (pigs)	*α*_2_	0.01–0.6	This work
Severity of density-dependent constraints (humans)	*α*_3_	0.001–0.1	This work
Rate of fecundity	*λ*	1 × 10^6^-1 × 10^7^	[61]
Rate of mortality of the adult parasite	*μ*_1_	0.33–1	[48, 58, 63]
Rate of egg mortality	*μ*_2_	50	[59, 60]
Rate of larvae mortality (pigs)	*μ*_3_	0.5–2	[15, 64]
Rate of larvae mortality (humans)	*μ*_4_	0.14–0.33	[14]

**Table 2:** Meaning of Variables and Parameters

Symbol	Variables	UNITS
*M*_1_	Mean intensity of taeniasis in humans	
*M*_2_	Mean intensity of cysticerci in the pigs	
*M*_3_	Mean intensity of cysticerci in humans	
*P*_1_	Prevalence of adult parasites (taenias)	
*P*_2_	Prevalence of larval parasites (cysticerci in pigs)	
*P*_3_	Prevalence of larval parasites (cysticerci in humans)	
*E*	Number of tapeworm eggs	
	PARAMETERS	
*k*_1_	Inverse measure of aggregation of T. solium	
*k*_2_	Inverse measure of aggregation of cysticerci	
*β*_1_	Rate of transmission of adult parasites	
*β*_2_	Rate of transmission of larvae to pigs	
*β*_3_	Rate of transmission of larvae to humans	
*D*_1_	Proportion of parasites acquired by the definitive host which survive to reach sexual maturity	
*D*_2_	Proportion of parasites acquired by the intermediate host which survive to reach infectivity	
*D*_3_	Proportion of parasites accidentally acquired by human host which survive to reach infectivity	
*T*_1_	Time period from entry into the definitive host to reproductive maturity when worms begin egg production	year
*T*_2_	Time period from entry into the intermediate host (pig) to the development of the infective stage	Year
*T*_3_	Time period from entry into the human host to the development of the infective stage	year
*H*_1_	Density of humans that can be infected with T. solium	
*H*_2_	Density of pigs that can be infected with cysticerci	
*H*_3_	Density of humans that can be infected with cysticerci	
*b*_1_	Rate of mortality of the definitive host (humans)	year^−1^
*b*_2_	Rate of mortality of the intermediate host (pigs)	year^−1^
*α*_1_	Severity of density-dependent constraints (taenia)	year^−1^
*α*_2_	Severity of density-dependent constraints (pigs)	year^−1^
*α*_3_	Severity of density-dependent constraints (humans)	year^−1^
*λ*	Rate of fecundity	year^−1^
*μ*_1_	Rate of mortality of the adult parasite	year^−1^
*μ*_2_	Rate of egg mortality	year^−1^
*μ*_3_	Rate of larvae mortality (pigs)	year^−1^
*μ*_4_	Rate of larvae mortality (humans)	year^−1^

### Bifurcation analysis of the parameters

**Fig. 3:**
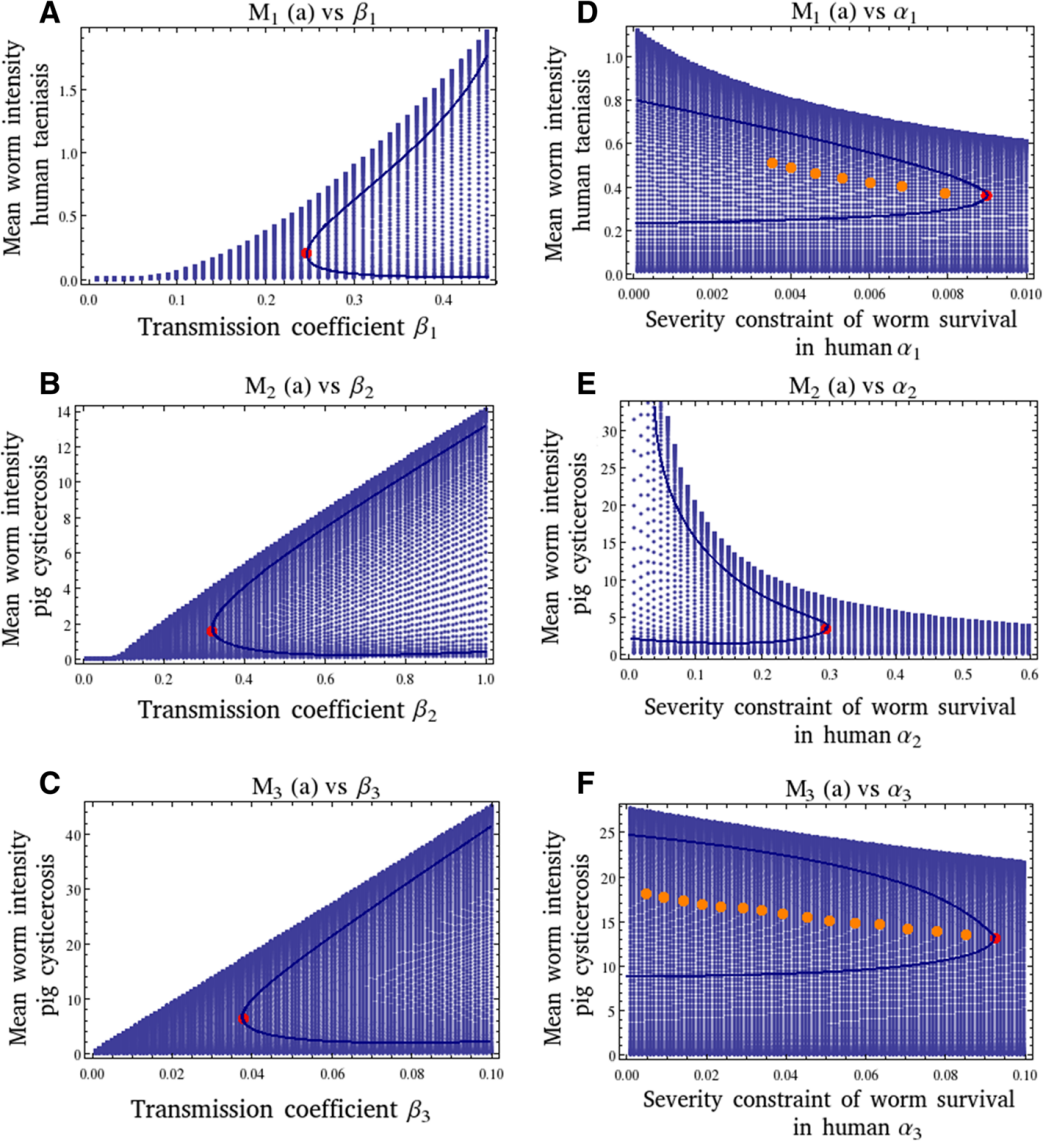
Bifurcation diagrams of the transmission coefficients (*β*_1_, *β*_2_, *β*_3_) and the severities of the density-dependent constraints (*α*_1_, *α*_2_, *α*_3_), as a function of their respective mean intensities (*M*_1_, *M*_2_, *M*_3_). The range of time is from 0 to 20 years with steps of 0.1. The values of the parameters are:*β*_1_ = 0.32 (except in A); *β*_2_ = 0.44 (except in B); *β*_3_ = 0.05 (except in C); *D*_1_ = 0.96; *D*_2_ = 0.79; *D*_3_ = 0.96; *H*_1_ = 0.001; *H*_2_ = 0.6; *b*_1_ = 0.016; *b*_2_ = 0.9; *α*_1_ = 0 : 001(except in D); *α*_2_ = 0.38 (except in E); *α*_3_ = 0.08 (except in F); *μ*_1_ = 1; *μ*_2_ = 50; *μ*_3_ = 1; *μ*_4_ = 0.33; *k*_1_ = 0.02; *k*_2_ = 0.3; *λ* = 1 × 10^7^; *c*_2_ = 0; *p*_2_ = 0; *N*_1_(0) = 1000; *N*_2_(0) = 1000. The value of *Ro* is 5.49. In all figures the solid lines denote the parabolic boundaries of the trajectories (see text for explanation). For each diagram, the breakpoints (*M*_*B*_), are marked as red dot. In D and F, there are multiple equilibria (marked in orange) until they reach a breakpoint. In D not all critical points are marked

In Fig. 3, bifurcation diagrams of the transmission coefficients (*β*_1_, *β*_2_, *β*_3_) and of the severities of the density-dependent constraints (*α*_1_, *α*_2_, *α*_3_), as a function of the mean intensities (*M*_1_, *M*_2_, *M*_3_), are presented. In all Figures, we show the equilibrium worm burden *M*^∗^(*a*) (the same can done with the equilibrium prevalence of infection *P*^∗^(*a*)) as a function of the transmission coefficients *β*_1_ (Fig. 3a), *β*_2_ (Fig. 3b), *β*_3_ (Fig. 3d), and the severities of the density-dependent constraints *α*_1_ (Fig. 3d), *α*_2_(Fig. 3e), and *α*_3_ (Fig. 3f). In all figures, the region of less density of points is the stable region. The enveloping curve shows the dynamic trajectory of *M*^∗^(*a*) following a perturbation from one of the two equilibrium states, *M*^∗^(*a*) endemic infection and *M*^∗^(*a*) = 0 (parasite extinction). In Fig. 3a, b and c, the upper branch is stable and as the parameters decreases, it reaches the unstable breakpoint *M*_*B*_, (marked with a red dot) which divides the upper branch from the lower which is unstable. Below these breakpoints, the transmission threshold *Ro*, the infection cannot be maintained. In Fig. 3a, b, and c note that there are positive relationships between the transmission coefficients of the infection in each type of host with their respective mean intensities (upper branch). This is particularly evident for values of *β*_1_ and *β*_2_ greater than 0.1. For values of *β*_1_ < 0.06 or *β*_2_ < 0.08, the value of *Ro* becomes less than unity. In Fig. 3a, note that *M*_1_ acquires the value of unity when *β*_1_ is of the order of 0.32. Therefore, a biological sensible range of values for *β*_1_ may lie between 0.1 and 0.36. In Fig. 3b, the value of *M*_2_ is not as restricted as *M*_1_, and therefore the range of values that *β*_2_ can acquire seems to lie between 0.35 and 1 considering the region of less density of points (stability) of the plot. Values of *β*_2_ » 1 may lead to superinfection in the pigs. In Fig. 3c, the same reasoning indicates that a likely range of values for *β*_3_ may be from very low transmission rates perhaps of the order of 0.036 up to 0.1.

In Fig. 3d, e, and f note that there are negative relationships between the magnitudes of the severities in each type of host with their respective mean intensities. In Fig. 3d, there are several enveloping curves, and therefore multiple stable equilibrium points which are so close that are difficult to highlight them all. In Fig. 3e, the upper branch is stable and as the parameter *α*_2_ increases, it reaches the unstable breakpoint *M*_*B*_, (marked with a red dot) which divides the upper branch from the lower which is unstable. In Fig. 3f, the dynamics of *M*^∗^(*a*) as a function of *α*_3_, display multiple stable equilibria with multiple breakpoints *M*_*B*_ (marked with red dots).

The rate of decrease of *M*_1_ and *M*_3_, as their corresponding values of severity increase, seems to be at a smaller pace than the rate of decay between *M*_2_ and *α*_2_. Considering that *M*_1_~1 the range of values for *α*_1_ seems to be very small lying between 0.001 and 0.01, and the range of values for *α*_3_ may be between 0.001 and 0.1. These results seem to be consistent with the common observation of mild immune responses of the human host against either *T. solium* or against cysticerci. This in turn, may be associated to a long coexistence between these developmental stages of the parasite within the human host. In contrast, the range of values of *α*_2_ seems to be larger (between 0.01 and 0.6) implying that cysticerci may be more sensible to immune responses mounted by pigs against them.

### Mean intensity and dynamics

**Fig. 4:**
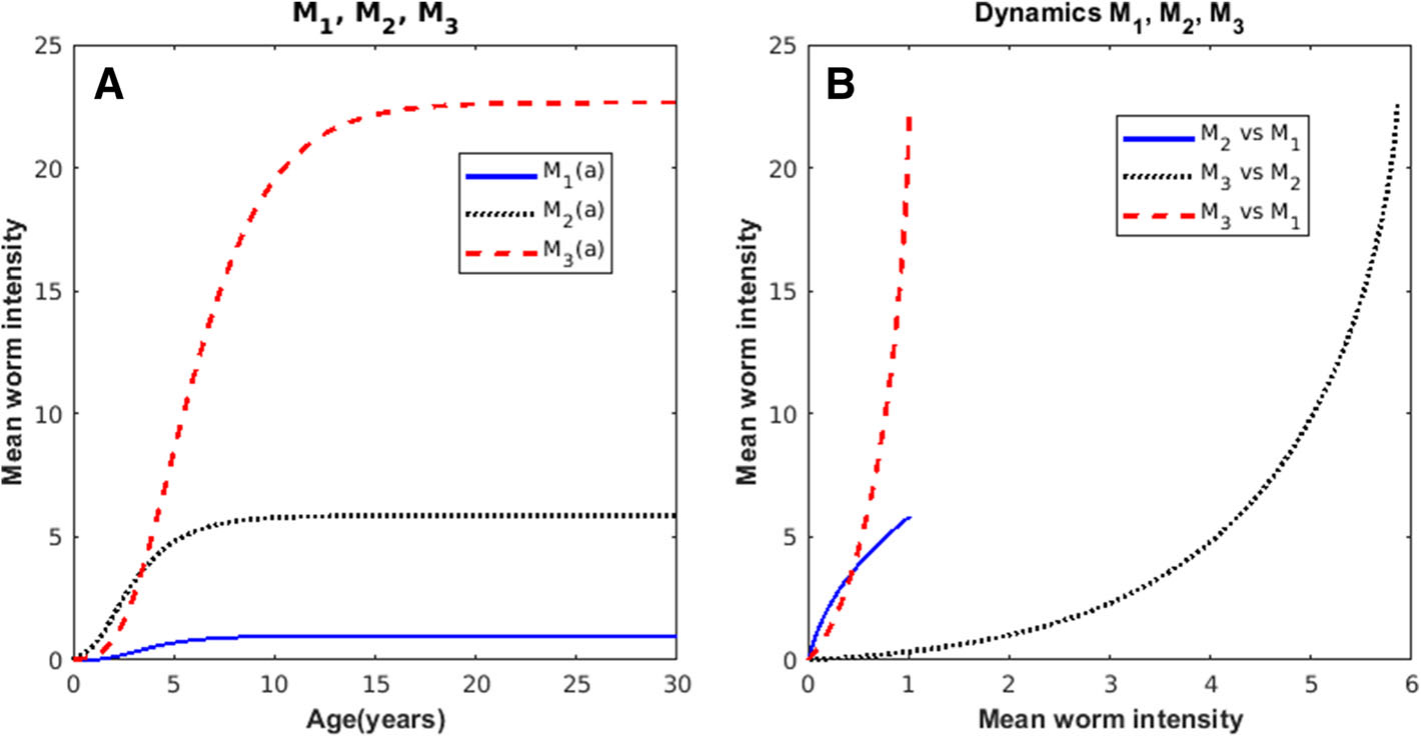
Computer simulated curves of the age-mean intensity of: **a** human taeniasis (blue-solid)*,* pig cysticercosis (black-dotted), and human cysticercosis (red-dashed) based upon Eqs. (4a), (4b), and (4c), respectively; **b** Phase spaces of the dynamics of: *M*_2_ versus *M*_3_ (solid), *M*_1_ versus *M*_3_ (dotted), and *M*_1_ versus *M*_2_ (dash-dotted). The values of the parameters are: *β*_1_ = 0.32; *β*_2_ = 0.44; *β*_3_ = 0.05; *D*_1_ = 0.96; *D*_2_ = 0.79; *D*_3_ = 0.96; *H*_1_ = 0.001; *H*_2_ = 0.6; *b*_1_ = 0.016; *b*_2_ = 0.9; *α*_1_ = 0.001; *α*_2_ = 0.38; *α*_3_ = 0.08; *μ*_1_ = 1; *μ*_2_ = 50; *μ*_3_ = 1; *μ*_4_ = 0.33; *k*_1_ = 0.02; *k*_2_ = 0.3; *λ* = 1 × 10^7^; *N*_1_(0) = 1000; *N*_2_(0) = 1000. The value of *Ro* is 5.49

In Fig. 4a a computer simulation of the mean worm burden of taenia (Eq. (4a)), of cysticerci in the pig (Eq. (4b)) and, cysticerci in humans (Eq. (4c)) as a function of age considering the range of values of the parameters given in Table 1 are shown. Note that the mean intensity of taenia rapidly reaches a plateau at *M*_1_~1. The age-mean intensities of pig and human cysticercosis follow a hyperbolic pattern. In Fig. 4b the phase diagrams of the mean intensities are shown. Note that *M*_2_ vs *M*_3_ (pig cysticercosis versus human cysticercosis) intersects with *M*_1_ vs *M*_2_ (human taeniasis versus pig cysticercosis) but not with *M*_1_ vs *M*_3_ (human taeniasis vs human cysticercosis).

## Mathematical model with chemotherapy

The mathematical model presented in the previous sections captures the essential aspects of the transmission dynamics of the infection of taenia-cysticercosis. This model is known in the literature of mathematical epidemiology as a density-dependent model that describes the flow of the parasite throughout the life cycle. Our interest now is to examine what could be the likely impact of the application of chemotherapy administered to pigs or humans upon the dynamics of the infection in pigs and humans. The derivation of the corresponding equations that allow for vaccination are presented in Part II. Effective and safe anthelminthic parasites are available for human taeniasis, pig cysticercosis, and human cystercosis such as praziquantel, niclosamide, albendazol, and oxfendazol [69–75].

The above model can be adapted to examine the long-term effects of the use of mass chemotherapy. When a drug is administered randomly within a population by treating a proportion G_1_ of the community per unit of time and the drug has an efficacy C_1_ (where C_1_ represents the average proportion of the worm burden killed by a single, or short course of treatment), the resulting increase in the per capita death rate of adult parasitic worms, *c*_1_, is [26, 28]:

6a $$ {c}_1=-\ln \left(1-{\mathrm{G}}_1\times {\mathrm{C}}_1\right) $$

The corresponding equation for the treatment against larval parasites in the pork population is,

6b $$ {c}_2=-\ln \left(1-{\mathrm{G}}_2\times {\mathrm{C}}_2\right) $$

Then Eqs. (4a), (4b) and (4c) are, respectively, the following:

7a $$ \frac{d{M}_1(a)}{da}={\beta}_1{D}_1{H}_2{M}_2(a)-\left({b}_1+{\mu}_1+{\alpha}_1-\ln \left(1-{\mathrm{G}}_1\times {\mathrm{C}}_1\right)\right){M}_1(a)-\frac{\alpha_1\left({k}_1+1\right){M}_1^2(a)}{k_1}, $$

7b $$ \frac{d{M}_2(a)}{da}=\frac{\beta_2{D}_2\lambda {H}_1{M}_1(a)}{\mu_2+{\beta}_2{H}_2+{\beta}_3{H}_3}-\left({\mu}_3+{b}_2+{\alpha}_2+{\beta}_1{H}_1-\ln \left(1-{\mathrm{G}}_2\times {\mathrm{C}}_2\right)\right){M}_2(a)-\frac{\alpha_2\left({k}_2+1\right){M}_2^2(a)}{k_2}, $$

7c $$ \frac{d{M}_3(a)}{da}=\frac{\beta_3{D}_3\lambda {H}_3{M}_1(a)}{\mu_2+{\beta}_2{H}_2+{\beta}_3{H}_3}-\Big({\mu}_4+{b}_1+{\alpha}_3-\ln \left(\left(1-{\mathrm{G}}_1\times {\mathrm{C}}_1\right)\right){M}_3(a)\operatorname{}. $$

## A susceptible-infected model of swine cysticercosis and human taeniasis

The previous model does not allow us to deal explicitly with hosts individuals that are susceptible, immune or vaccinated. The model refers to the worm loads of already infected individuals. Herein we extend the previous model to allow for the evaluation of different community interventions: application of a vaccine against either human taeniasis and/or swine cysticercosis and/or chemotherapeutic interventions against either the taenia and/or the larval cysticerci.

Let us denote the size of pig population as *N*_2_ = *J*_2_ + *S*_2_,where *J*_2_ is the number of infected pigs, *S*_2_ is the number of susceptible pigs. Considering that the distribution of cysticerci among the pigs in a community can be described by a negative binomial distribution, the relationship between the proportion of infected individuals and the mean worm burden is,

8 $$ \frac{J_2}{N_2}=1-{\left(1+\frac{M_2(a)}{k_2}\right)}^{-{k}_2}=1-{\hat{S}}_2(a), $$

where ${\hat{S}}_2(a)$ denotes the proportion of susceptible pigs at age *a*. Then the number of susceptible pigs at age *a* can be expressed as,

9 $$ {S}_2(a)={N}_2(a){\left(1+\frac{M_2(a)}{k_2}\right)}^{-{k}_2}. $$

If *N*_2_(*a*) = *N*_2_(0) exp(−*b*_2_*a*) where *N*_2_(0) is the initial population size of pigs, it follows that the rate of change of the number of susceptible individuals becomes,

10 $$ \frac{d{S}_2(a)}{da}=-{S}_2(a)\left[{\left(1+\frac{M_2(a)}{k_2}\right)}^{-1}\frac{d{M}_2(a)}{da}+{b}_2\right]. $$

The equations that describe the rate of change of susceptibles to human taeniasis (*S*_1_) and the rate of change of susceptibles to human cysticercosis (*S*_3_) are, respectively,

11 $$ \frac{d{S}_1(a)}{da}=-{S}_1(a)\left[{\left(1+\frac{M_1(a)}{k_1}\right)}^{-1}\frac{d{M}_1(a)}{da}+{b}_1\right] $$

12 $$ \frac{dS_3(a)}{da}=-{b}_1{N}_1(0)\exp \left(-{b}_1a\right)-{H}_3\left[\frac{dM_3(a)}{da}\right], $$

where *N*_1_(0) is the initial human population size. The corresponding equations for describing the prevalence of human taeniasis (*J*_1_), the prevalence of pig cysticercosis (*J*_2_), and the prevalence of human cysticercosis (*J*_3_) are,

13 $$ \frac{d{J}_1(a)}{da}=-{b}_1{N}_1(0)\exp \left(-{b}_1a\right)-\frac{d{S}_1(a)}{da} $$

14 $$ \frac{d{J}_2(a)}{da}=-{b}_2{N}_2(0)\exp \left(-{b}_2a\right)-\frac{d{S}_2(a)}{da} $$

15 $$ \frac{d{J}_3(a)}{da}={H}_3\frac{d{M}_3(a)}{da} $$

It is worthwhile to mention that *Ro* in this SI model (Eqs. 10–15) is given by Eq. 5.

## Results

### Computer simulation experiments of different chemotherapeutic strategies

#### Density-dependent model

**Fig. 5:**
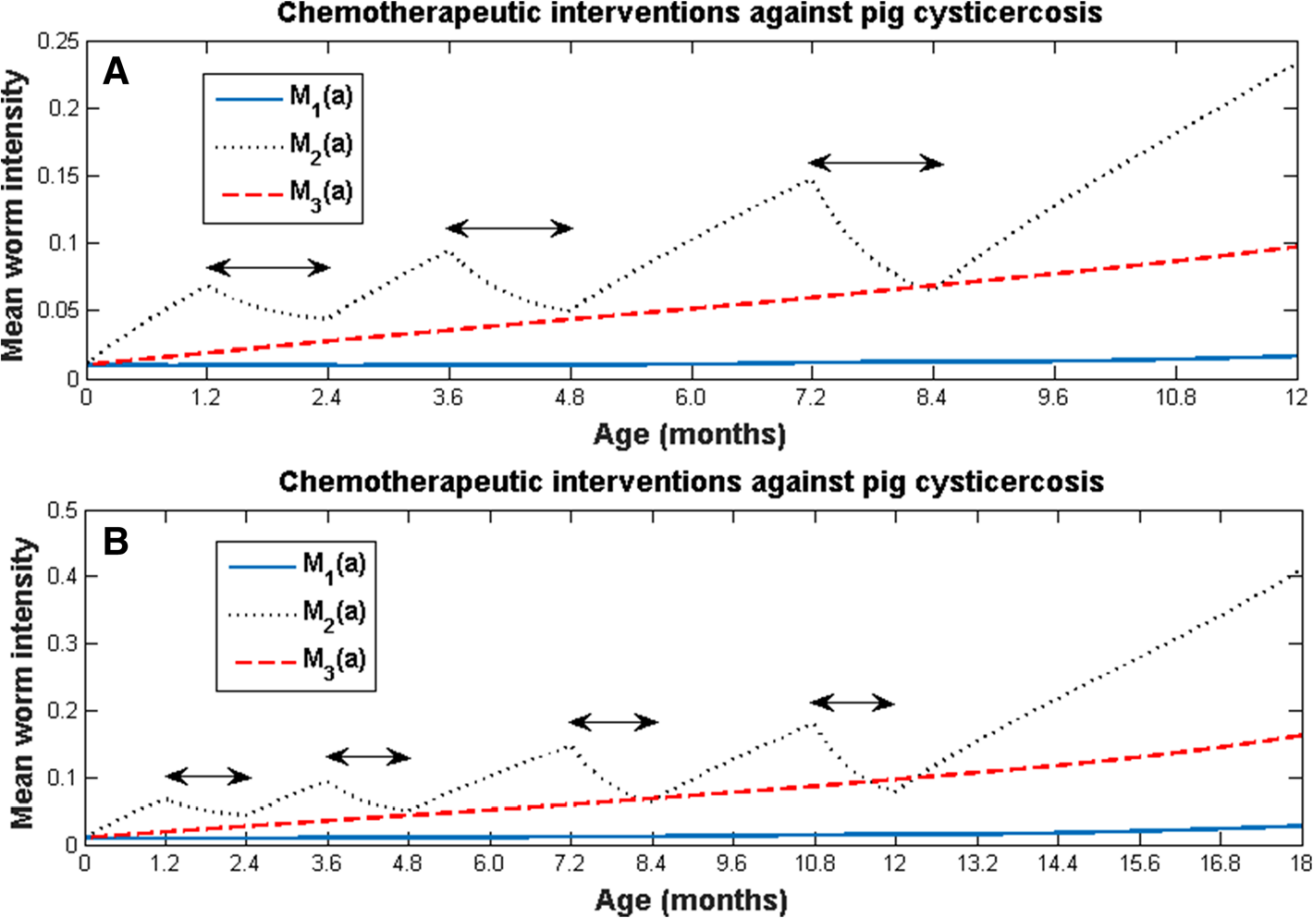
Chemotherapeutic interventions against pig cysticercosis: **a** from 1.2 to 2.4 months of age (first double arrow), no intervention during 1 month, intervention again but to pigs from 3.6 to 4.8 months (second double arrow), no intervention during 2 months, and intervention to pigs from 7.2 to 8.4 months of age (third double arrow), consecutively; **b** The treatment schedule is the same as that shown in (**a**), but after no intervention from 8.4 to 10.8 months, there is an additional treatment to pigs of 10.8 to 12 months of age (fourth double arrow). The values of the parameters are the same as those used in Fig. 4, and G_2_ = 0.99; C_2_ = 0.99.

To illustrate accessible chemotherapeutic interventions, we show the results of applying mass chemotherapy only to pigs as follows: from 1.2 to 2.4 months of age, no intervention during 1 month, intervention again but to pigs from 3.6 to 4.8 months, no intervention during 2 months, and intervention to pigs from 7.2 to 8.4 months of age, consecutively (Fig. 5a); The other treatment schedule is the same as that shown in Fig. 5a, but there is no intervention from 8.4 to 10.8 months, and an additional treatment is applied to pigs of 10.8 to 12 months of age (Fig. 5b). In both cases, the rate of increase of the mean worm intensity is always reduced after the application of the drug, but after the treatment ceases, the mean worm burden increases again, and an increasing saw-toothed pattern emerges in both cases. After the treatment ceases the mean worm burden continues to increase as if no treatments had been applied. We also note that the greatest reduction in the mean worm burden is achieved in the second chemotherapeutic regime (Fig. 5b) when the drug is administered to pigs from 10.8 to 12 months of age. The latter coincides with the moment in which the infection starts to stabilize (see Fig. 4). These computer experiments highlight the robustness of the endemicity of pig cysticercosis. The effect of these chemotherapeutic regimes on human taeniasis and human cysticercosis is simply to delay the rate at which they tend to stabilize their mean worm burdens as a function of age. Computer simulations against human taeniasis with different values of G_1_ and C_1_ are found in Additional file 2.

## Compartmental model

**Fig. 6:**
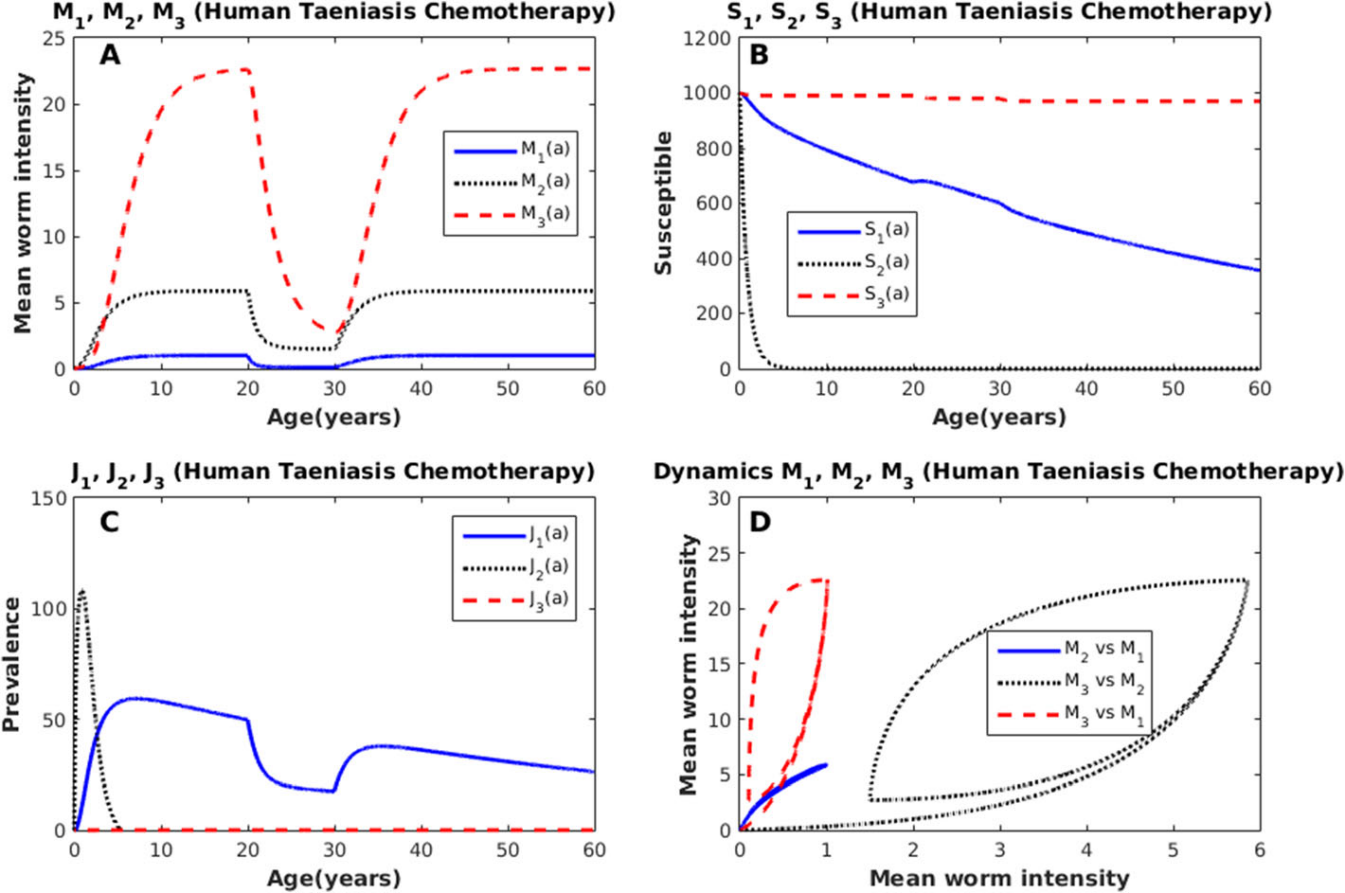
Chemotherapeutic intervention against human taeniasis (blue-solid); pig cysticercosis (black-dotted); human cysticercosis (red-dashed). **a** Mean worm intensities (**b**) Age-susceptibles (**c**) Age-prevalence (**d**) Phase spaces of the dynamics: *M*_3_ versus *M*_2_ (red-dashed), *M*_3_ versus *M*_1_ (blue-dotted), and *M*_2_ versus *M*_1_ (black-solid). The values of the parameters are: *β*_1_ = 0.32; *β*_2_ = 0.44; *β*_3_ = 0.05; *D*_1_ = 0.96; *D*_2_ = 0.79; *D*_3_ = 0.96; *H*_1_ = 0.001; *H*_2_ = 0.6; *H*_3_ = 0.001; *b*_1_ = 0.016; *b*_2_ = 0.9; *α*_1_ = 0.001; *α*_2_ = 0.38; *α*_3_ = 0.08; *μ*_1_ = 1; *μ*_2_ = 50; *μ*_3_ = 1; *μ*_4_ = 0.33; *k*_1_ = 0.02; *k*_2_ = 0.3; *λ* = 1 × 10^7^; G_1_ = 0.9; C_1_ = 0.9; *N*_1_(0) = 1000; *N*_2_(0) = 1000. The value of *Ro* is 5.49

Herein, we explore the dynamics of the SI model by simulating a chemotherapeutic mass treatment to infected humans with taeniasis. The treatment is administered to 90% of infected individuals, during a period of 10 years, and considering 90% drug efficacy. Notice that the mean intensities, not only of human taeniasis, but also of human cysticercosis and pig cysticercosis are reduced after the application of the anthelmintic (Fig. 6a). After the intervention ceases the mean intensities return to their pre-control values as has been reported for other helminth infections [25, 26, 76, 77]. In this computer simulation experiment, it turns out that the best strategy would be to treat individuals with human taeniasis since it has the greatest effect in the reduction of mean intensities. However, this result cannot be generalized since other parameter values indicate that the best strategy could be to tackle pig cysticercosis only. It is also shown that the reduction of the mean worm burden of pig cysticercosis and/or human taeniasis improves the health conditions of other hosts. The number of susceptible individuals to human taeniasis (*S*_1_) and the number of susceptible individuals to human cysticercosis (*S*_3_) decrease linearly and monotonically as a function of age, whilst the number of susceptible animals to porcine cysticercosis (*S*_2_) follows an exponential decay (Fig. 6b). The prevalence of human taeniasis infection (*J*_1_) increases rapidly with age but its prevalence is reduced while the treatment is applied (Fig. 6c). A similar pattern has been observed in natural communities [52]. The prevalence of infection of pig cysticercosis (*J*_2_) increases during the first months of the life of the pigs and eventually declines to zero (Fig. 6c). The prevalence of pig cysticercosis goes to zero before the first year of life of the pigs (Fig. 6c). We emphasize that we are dealing with a Susceptible-Infected model in which a compartment of recovered individuals is not included. The prevalence of infection of human cysticercosis (*J*_3_) is maintained at very low values (Fig. 6c) as has been observed in natural communities [78]. The dynamics is shown in Fig. 6d. Note the large excursions of the trajectories of *M*_3_ versus *M*_2_, *M*_3_ versus *M*_1_, and the short trajectory of *M*_2_ versus *M*_1_.

## Discussion

In this work, we present a mathematical model of the transmission dynamics of taeniasis-cysticercosis based upon the life cycle of the parasite. This model consists of a set of three differential equations, which are density-dependent equations that describe the flow of the parasite through the definitive and the intermediate host as well as humans infected with cysticercosis. Empirical evidence of the transmission dynamics of taeniasis-cysticercosis is analysed and is used to formulate the present model. The values of the parameters of the model were chosen from the literature and others, such as the degree of aggregation of taenia, were deduced from the fact that most of the time only one or very few tapeworms are present per individual. The stability behaviour of the mean intensities as a function of the transmission coefficients (*β*_1_, *β*_2_, *β*_3_) and of the severities of the density-dependent constraints (*α*_1_, *α*_2_, *α*_3_), were explored by means of bifurcation diagrams. Both the bifurcation analyses and the sensitivity analysis led to feasible ranges of values of these 6 parameters. The model mimics the observed patterns of human taeniasis, pig and human cysticercosis. For example, for a wide range of combination of values of the parameters given in Table 1, the mean intensity of adult worms tends to rapidly stabilize in one parasite per individual host. Therefore, the natural adult worm mortality becomes density independent. The model initially includes two continuous dynamic variables: one for age (a), and one for time (t). A simplification is performed to focus only on the dynamic variable for age and ignoring time. This is equivalent to a time-age advection model and setting the time derivative to zero. We found rich dynamics of the age-dependent model like multiple equilibria, stable and unstable regions, breakpoints and the threshold *Ro*. However, several improvements can be made to the present model: to include the behaviour over time and age, and to make the state variables, like the mean intensity, prevalence and, susceptibles, random variables. The latter improvements will certainly strengthen or weaken our predictions, particularly those related to the control and/or eventual elimination of the infection. In the context of our model, the reduction of the number of relevant parameters and the explicit incorporation of both acquired immunity and behavioural ecology is completely feasible [30]. One of the hallmarks of the immunology of taeniid cestode infection in their intermediate hosts is that infected hosts are immune to re-infection [79]. This situation is referred to as concomitant immunity, i.e., an infected animal is immune to re-infection, while at the same time parasites from the initial infection remain unaffected. Immunity wanes irrespective of the continued presence of mature metacestodes.

One novelty of our model is that it fills gaps of lack of knowledge about the parasites transmission dynamics and the effect of available intervention tools (see also Part 2). Our model also calls for further experimental work in naturally infected pigs to determine what is the initial level of infection that leads to immunity and the duration of immunity.

One of the most distinctive features of helminth infections is that they are very stable to climatic or environmental or man-made perturbations [24–26, 28, 29, 33–35, 76, 77]. Most helminth models developed so far deal with control measures such as the application of chemotherapy. The typical result is that after the application of chemotherapy the mean intensity of the helminth parasite returns to their pre-control levels of infection [76, 77]. Our CSE show a case in which chemotherapy against adult worms is more effective than the chemotherapy against the cysticerci. We also obtained cases in which chemotherapy against pig cysticercosis is more effective than the chemotherapy against human teniasisis (not shown).

The most significant conclusion to be drawn from the quantitative analysis of the CSE is the following: Chemotherapeutic interventions against pig cysticercosis or against human taeniasis may reduce rapidly and effectively the mean intensity of human taeniasis, pig cysticercosis and human cysticercosis. This effect can be achieved even if the protective efficacy of the drug is of the order of 90% and the coverage rate is also of the order of 90%. This means that health in humans infected either with taenias or cysticerci may be achieved by the application of anthelmintic drugs against pig cysticercosis.

From the same life cycle, humans are infected with cysticercosis accidentally. Our results show that if the goal of the program is to reduce the burden of epilepsy associated to neurocysticercosis it may be possible to achieve substantial reductions if chemotherapy is applied to human cysticercosis only. We also observed that the number of susceptible individuals as a function of age to human cysticercosis is always a negative straight line since the value of *Ro* does not practically contribute to this number except for the term *β*_3_*H*_3_ in the denominator of Eq. (5).

The density-dependent model was extended to a Susceptible-Infected (SI) model which comprises compartmental equations that describe susceptible and infected hosts. This SI model can include vaccination of susceptibles against either human taeniasis and/or against pig cysticercosis. When vaccination strategies against either swine cysticercosis and/or human taeniasis are incorporated to the basic model, new differential equations are needed to identify infected, susceptible, and vaccinated individuals (Part 2). Then, the present model consists of six compartmental equations, one for susceptibles and the other for infected, for each of the three hosts. The resulting mathematical model is then a coupled system of non-linear differential equations, some of which are density-dependent equations and others are compartmental equations. The rationale of the present model is to build a bridge between density-dependent equations with compartmental equations to describe the dynamics of all the hosts that participate in the life cycle of the parasite. This approach can be used for other helminth infections either of direct or indirect transmission (ascaris, schistosomes, hookworms, etcetera). The modification of these equations to include vaccinated individuals is presented in Part II. Then we can assess whether the impact of different vaccination strategies impinge either on the mean intensity and/or the prevalence of the infection and/or the population of susceptible individuals of different hosts. The relative contributions of chemotherapy (and vaccination) can be safely evaluated.

Any mathematical model, no matter how complicated, consists of a set of assumptions, from which a set of conclusions are deduced. Provided the model is correct, if we accept its assumptions, we must by logic also accept its conclusions. A mathematical model is a logical machine for converting assumptions into conclusions. The conclusions are already wrapped up into the assumptions, of which they are a logical consequence. This is not to say that the conclusions are obvious. Instead of asking whether we accept the conclusions of the model, we should be asking whether we accept the model’s assumptions.

It has been suggested that if a vaccine for cestodes were available and if the immunity changed rapidly, then the endemic steady state may be destabilized, resulting in oscillations in the parasite population [80]. In our CSE, the inspection of the phase spaces does not seem to support that notion.

No effective density-dependent constraints were observed for *T. hydatigena* and *T. ovis* in the definitive host [80], except the one in the form of acquired immunity in the intermediate host.

Then the combination of administering anti-helminthic drugs to individual suffering from taeniasis and administering the vaccine to pigs against the infection with cysticerci to prevent and even to eliminate this parasite disease is examined in Part II. The recommendations of the International Task Force for Disease Eradication in 1993 placed cysticercosis among 6 potentially eradicable diseases [81], and more recently The World Health Organization has placed this infection as one of the neglected diseases to overcome by integrated approaches [82, 83]. We hope that the present work may contribute to that target. Finally, we stress that our model contains no arbitrarily adjustable or unmeasurable parameters, it is consistent with both the biology of the life cycle of the parasite and with observed patterns in the field, and it successfully predicts a variety of interesting and unexpected phenomena.

## Conclusions

We developed the first density-dependent model based upon the actual transmission dynamics of taeniasis-cysticercosis, and one of the first models of helminth parasites that allows the evaluation of the impact of chemotherapeutic interventions upon all hosts involved in the life cycle of the parasite even those that are accidentally infected. From the density-dependent model, we directly derive a susceptible-infected compartmental model with which the impact of vaccination strategies can be evaluated. The whole model represents a significant advance when compared to previous models of taeniasis-cysticercosis.

## Additional files

Additional file 1: Stabilty Analysis. (PDF 35 kb)

Additional file 2: Chemotherapeutic interventions against human taeniasis with different drug efficacies and coverage rates. (PDF 397 kb)
