# Supplementary material for: Mathematical model of the life cycle of taenia-cysticercosis: transmission dynamics and chemotherapy (Part 1)
Source: Theor Biol Med Model. 2018 Nov 19;15:18. doi: 10.1186/s12976-018-0090-0 (PMC6241031; doi:10.1186/s12976-018-0090-0)
Supplement: Supplementary file 1 — Stabilty Analysis. (PDF 35 kb) [file 12976_2018_90_MOESM1_ESM.pdf]

SUPPLEMENTARY MATERIAL 1

Stabilty Analysis

We rewrite Eqs (4a), 4(b) and 4(c) as,

$$\begin{aligned}\frac{dM_1(a)}{\partial a} &= AM_2(a) + BM_1(a) = F_1(M_1, M_2, M_3)\\ \frac{dM_2(a)}{\partial a} &= DM_1(a) + EM_2(a) = F_2(M_1, M_2, M_3)\\ \frac{dM_3(a)}{\partial a} &= FM_1(a) + GM_3(a) = F_3(M_1, M_2, M_3)\end{aligned} \qquad \text{(A1)}$$

where $A = \beta_1 D_1 H_2$; $B = -(b_1 + \mu_1)$; $D = \dfrac{\beta_2 D_2 \lambda H_1}{\mu_2 + \beta_2 H_2 + \beta_3 H_3}$; $E = -(\mu_3 + b_2 + \beta_1 H_1)$; $F = \dfrac{\beta_3 D_3 \lambda H_1}{\mu_2 + \beta_2 H_2 + \beta_3 H_3}$; $G = -(b_1 + \mu_4)$. We want to find the elements a_{ij} of the Jacobian matrix from the prescription $a_{ij} = \left(\dfrac{\partial F_i}{\partial M_j}\right)^*$. Then, the Jacobian matrix is,

$$J = \begin{vmatrix} B-\lambda & A & 0 \\ D & E-\lambda & 0 \\ F & 0 & G-\lambda \end{vmatrix} = (B-\lambda)(E-\lambda)(G-\lambda) - AD(G-\lambda) = 0$$

The characteristic equation is, $|B-\lambda| = (B-\lambda)(E-\lambda)(G-\lambda) - AD(G-\lambda) = 0$. Rearranging terms,

$$\begin{aligned}&-\lambda^3 + (B+G+E)\lambda^2 + (-BE - BG - EG + AD)\lambda - ADG + BEG = 0\\ &\lambda^3 - (B+G+E)\lambda^2 + (BE + BG + EG - AD)\lambda + ADG - BEG = 0\end{aligned}$$

If J is an $m \times m$ matrix, the equation $\det|J - \lambda I| = 0$ for the eigenvalues λ comes down to an mth – order polynomial equation,

$$\lambda^m + a_1\lambda^{m-1} + a_2\lambda^{m-2} + \cdots + a_m = 0$$

The Routh-Hurwitz criterion gives constraints on the coefficients $a_1, a_2, ..., a_m$ which are necessary and sufficient to ensure all eigenvalues lie in the left-hand complex plane. The stability conditions for $m = 3$ are:

$$a_1 > 0; a_3 > 0; a_1 a_2 > a_3 \qquad \text{(A2)}$$

First, we must show that $a_1 > 0$, i.e., $-(B+G+E) > 0$. Substituting the values for each constant, we obtain:

$$-\left(-\left(b_1+\mu_1\right)-\left(b_2+\mu_3+\beta_1 H_1\right)-\left(\mu_4+b_1\right)\right) > 0 \Rightarrow$$
$$\left(b_1+\mu_1\right)+\left(b_2+\mu_3+\beta_1 H_1\right)+\left(\mu_4+b_1\right) > 0$$

As all constants are positive then $a_1 > 0$.

The second condition is $a_3 > 0$, i.e., $(ADG - BEG) > 0$; rearranging, $G(AD - BE) > 0$; then from Eq. X we obtain $G = -(\mu_3 + b_1)$ since the 2 constants are positive. Now, let see what happens with $AD - BE$:

$$AD - BE = \left(\beta_1 D_1 H_2\right)\left(\frac{\beta_2 D_2 \lambda H_1}{\mu_2+\beta_2 H_2+\beta_3 H_3}\right)-\left(-\left(b_1+\mu_1\right)\right)\left(-\left(b_2+\mu_3+\beta_1 H_1\right)\right) > 0$$
$$\Rightarrow \frac{\beta_1 D_1 H_2 \beta_2 D_2 \lambda H_1}{\mu_2+\beta_2 H_2+\beta_3 H_3} > \left(b_1+\mu_1\right)\left(b_2+\mu_3+\beta_1 H_1\right)$$
$$\Rightarrow \frac{\beta_1 D_1 H_2 \beta_2 D_2 \lambda H_1}{\left(\mu_2+\beta_2 H_2+\beta_3 H_3\right)\left(b_1+\mu_1\right)\left(b_2+\mu_3+\beta_1 H_1\right)} > 1$$

Then, the basic reproductive number is:

$$R_0 = \frac{\beta_1 D_1 H_2 \beta_2 D_2 \lambda H_1}{\left(\mu_2+\beta_2 H_2+\beta_3 H_3\right)\left(b_1+\mu_1\right)\left(b_2+\mu_3+\beta_1 H_1\right)}$$

If $R_0 > 1$ the parasite will endemically persist in the population and hence $a_3 > 0$.

Finally, we have to show that $a_1 a_2 > a_3$. We have already shown that $a_1 > 0$ and $a_3 > 0$. If we show that $a_2 > 0$ then we could conclude that $a_1 a_2 > a_3$.

$$a_2 > 0 \Rightarrow BE + G\left(B+E\right) - AD > 0 \Rightarrow BE + G\left(B+E\right) > AD \Rightarrow,$$
$$\frac{BE + G\left(B+E\right)}{AD} > 1$$

Then, $BE > 0$, $G < 0$, and $B + E < 0 \Rightarrow G(B+E) > 0 : A > 0$ and $D > 0 \Rightarrow AD > 0$. Then it follows that $\frac{BE + G(B+E)}{AD} > 1$. Therefore $a_2 > 0 \Rightarrow a_1 a_2 > a_3$. Then the system (A1) fulfills all conditions of stability (A2).
