# Supplementary material for: Mathematical model of the life cycle of taenia-cysticercosis: transmission dynamics and chemotherapy (Part 1)
Source: Theor Biol Med Model. 2018 Nov 19;15:18. doi: 10.1186/s12976-018-0090-0 (PMC6241031; doi:10.1186/s12976-018-0090-0)
Supplement: Supplementary file 2 — Chemotherapeutic interventions against human taeniasis with different drug efficacies and coverage rates. (PDF 397 kb) [file 12976_2018_90_MOESM2_ESM.pdf]

SUPPLEMENTARY MATERIAL 2

Chemotherapeutic interventions against human taeniasis with different drug efficacies and coverage rates

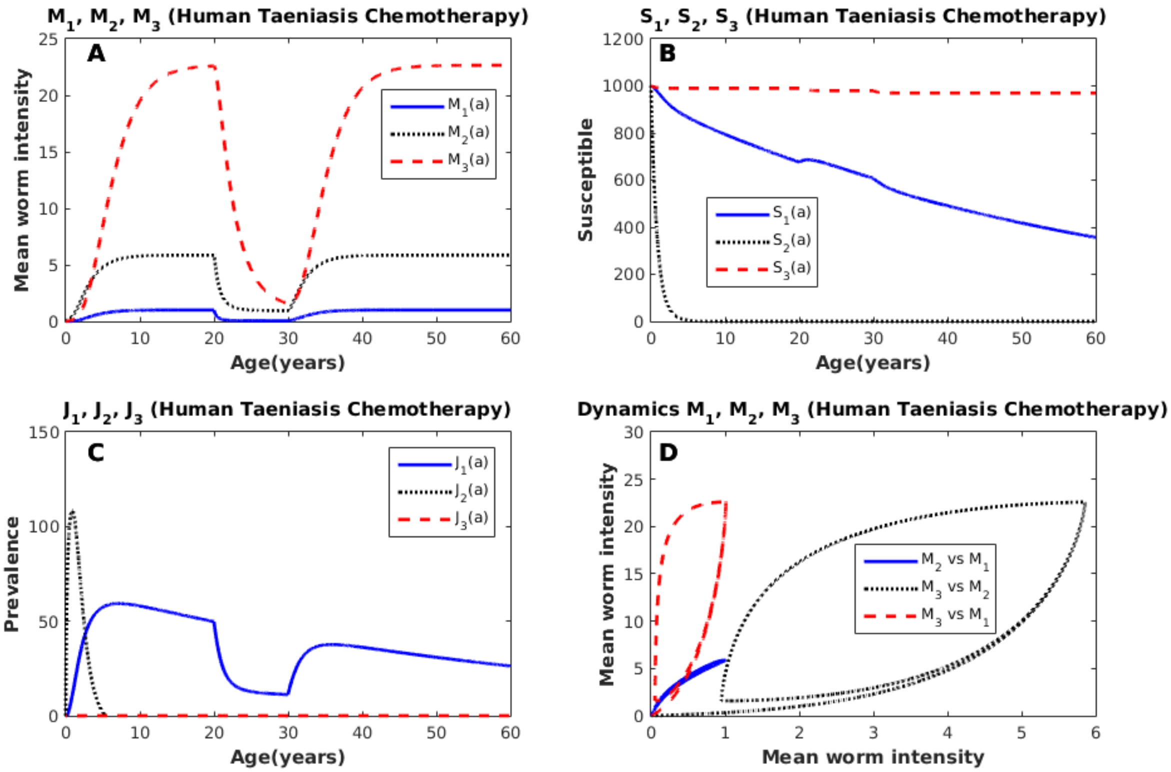

Figure 1S. Chemotherapeutic intervention against human taeniasis (blue-solid), pig cysticercosis (black-dotted) and, human cysticercosis (red-dashed). (A) Mean worm intensities (B) Age-susceptibles (C) Age-prevalence (D) Phase spaces of the dynamics: M_3 versus M_2 (black-dotted), M_3 versus M_1 (red-dashed), and M_2 versus M_1 (blue-solid). The values of the parameters are the same as those used in **Fig. 6**, and $G_1 = 100$ and $C_1 = 90$.

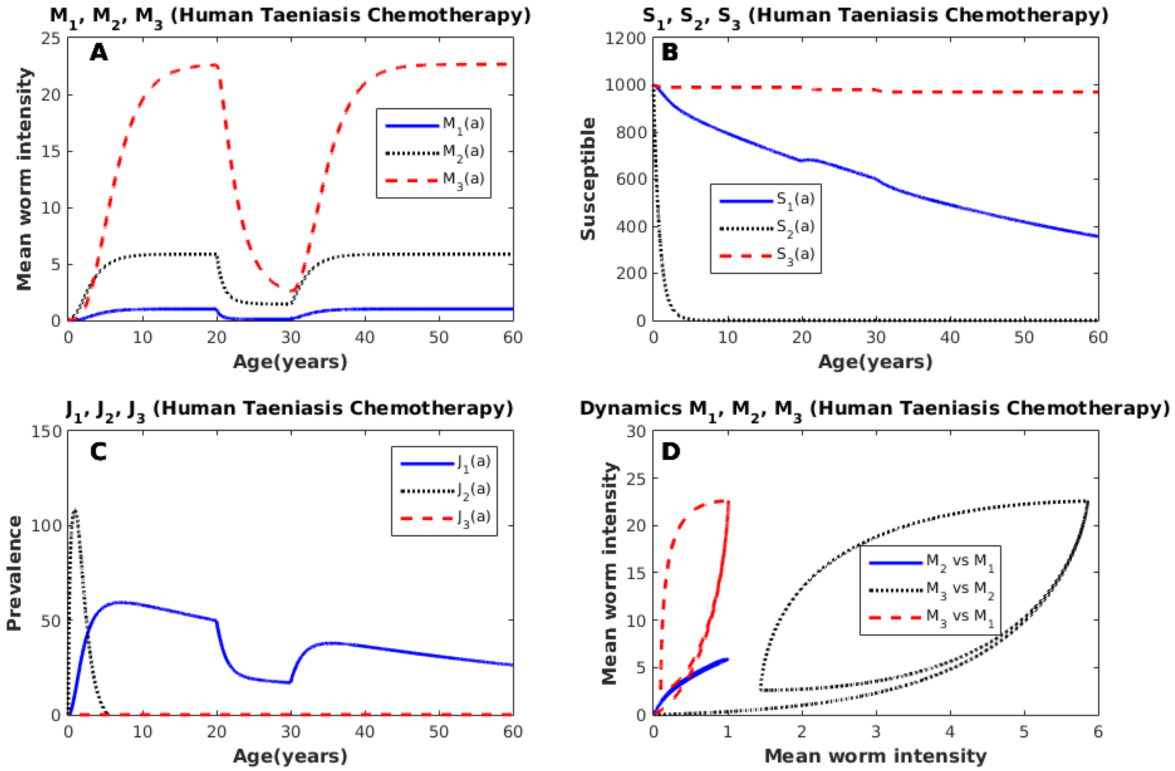

Figure 2S. Chemotherapeutic intervention against human taeniasis (blue-solid), pig cysticercosis (black-dotted) and, human cysticercosis (red-dashed). (A) Mean worm intensities (B) Age-susceptibles (C) Age-prevalence (D) Phase spaces of the dynamics: M_3 versus M_2 (black-dotted), M_3 versus M_1 (red-dashed), and M_2 versus M_1 (blue-solid). The values of the parameters are the same as those used in **Fig. 6**, and $G_1 = 100$ and $C_1 = 80$.

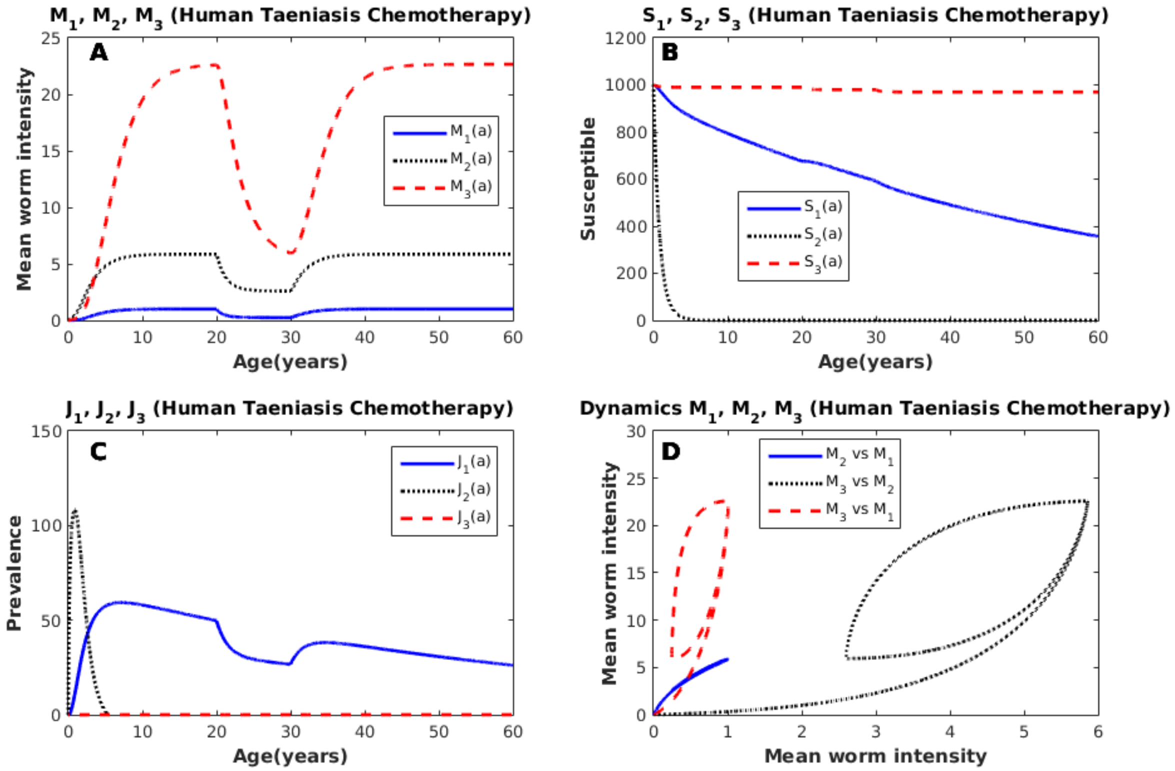

Figure 3S. Chemotherapeutic intervention against human taeniasis (blue-solid), pig cysticercosis (black-dotted) and, human cysticercosis (red-dashed). (A) Mean worm intensities (B) Age-susceptibles (C) Age-prevalence (D) Phase spaces of the dynamics: M_3 versus M_2 (black-dotted), M_3 versus M_1 (red-dashed), and M_2 versus M_1 (blue-solid). The values of the parameters are the same as those used in **Fig. 6**, and $G_1 = 100$ and $C_1 = 60$.

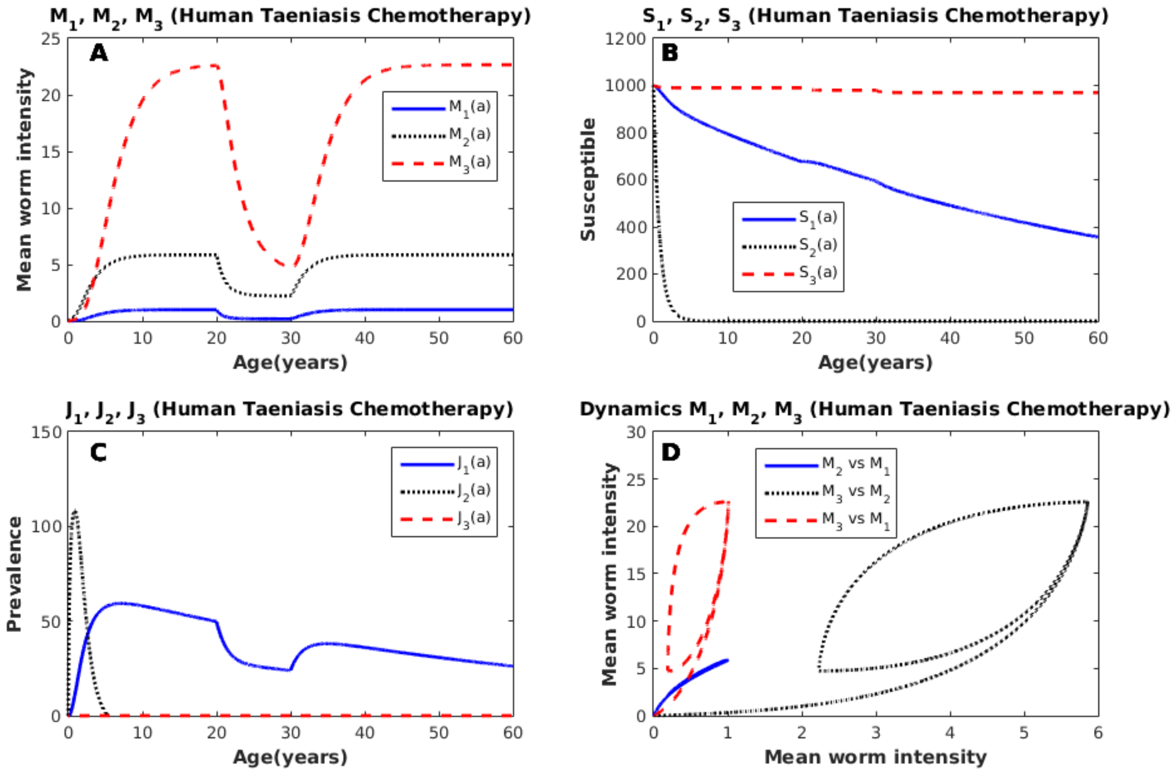

Figure 4S. Chemotherapeutic intervention against human taeniasis (blue-solid), pig cysticercosis (black-dotted) and, human cysticercosis (red-dashed). (A) Mean worm intensities (B) Age-susceptibles (C) Age-prevalence (D) Phase spaces of the dynamics: M_3 versus M_2 (black-dotted), M_3 versus M_1 (red-dashed), and M_2 versus M_1 (blue-solid). The values of the parameters are the same as those used in **Fig. 6**, and $G_1 = 80$ and $C_1 = 80$.

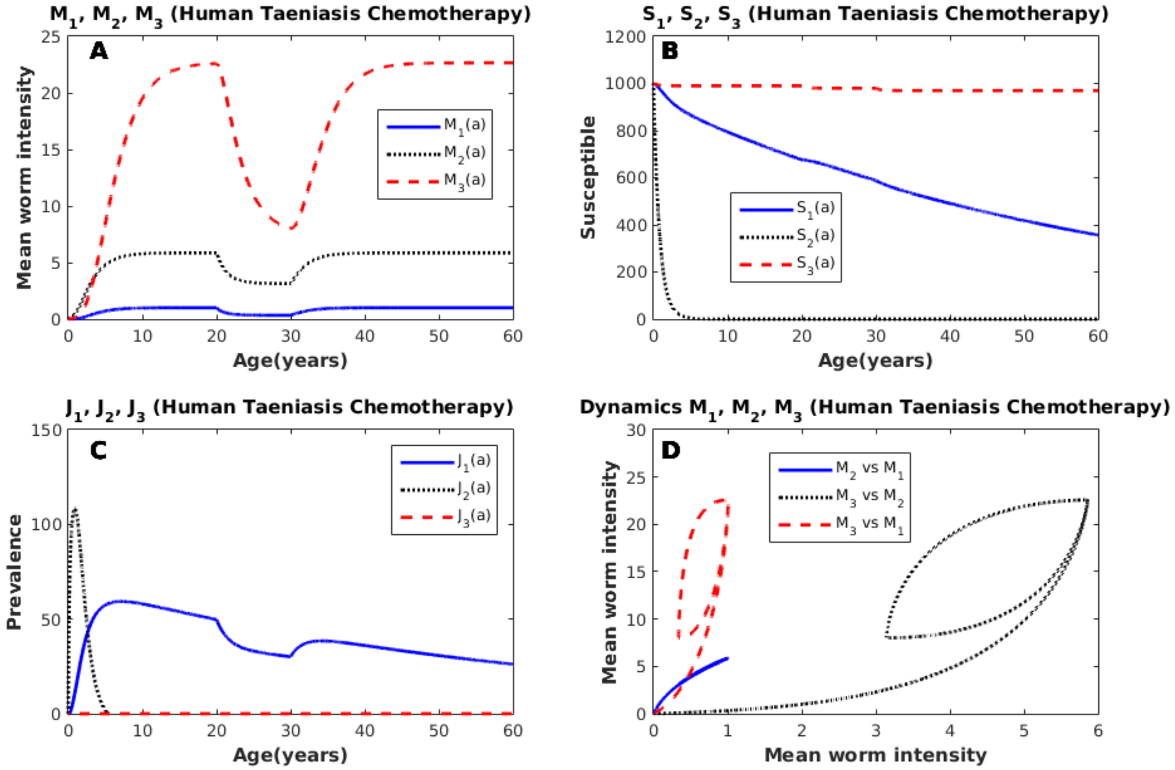

Figure 5S. Chemotherapeutic intervention against human taeniasis (blue-solid), pig cysticercosis (black-dotted) and, human cysticercosis (red-dashed). (A) Mean worm intensities (B) Age-susceptibles (C) Age-prevalence (D) Phase spaces of the dynamics: M_3 versus M_2 (black-dotted), M_3 versus M_1 (red-dashed), and M_2 versus M_1 (blue-solid). The values of the parameters are the same as those used in **Fig. 6**, and $G_1 = 80$ and $C_1 = 60$.

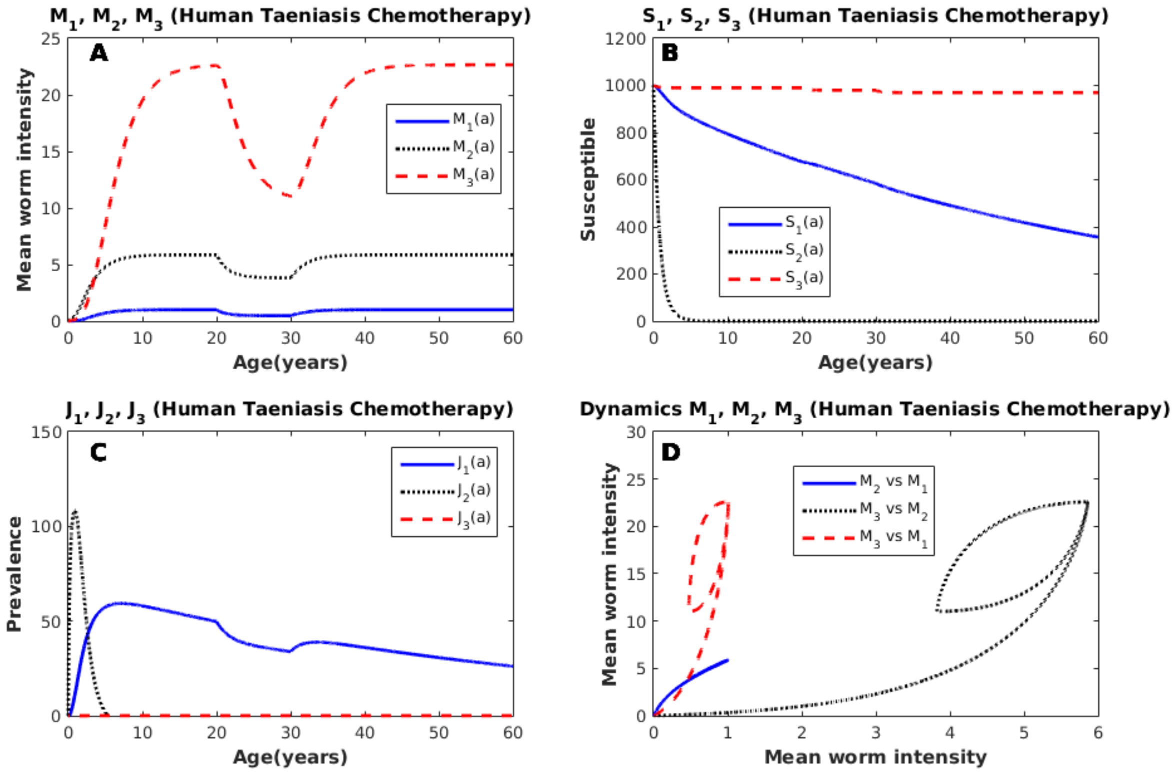

Figure 6S. Chemotherapeutic intervention against human taeniasis (blue-solid), pig cysticercosis (black-dotted) and, human cysticercosis (red-dashed). (A) Mean worm intensities (B) Age-susceptibles (C) Age-prevalence (D) Phase spaces of the dynamics: M_3 versus M_2 (black-dotted), M_3 versus M_1 (red-dashed), and M_2 versus M_1 (blue-solid). The values of the parameters are the same as those used in **Fig. 6**, and $G_1 = 60$ and $C_1 = 60$.
